# Molecular targeted therapy in combination with chemotherapy for the treatment of platinum-resistant/refractory ovarian cancer (PROC): a systematic review and network meta-analysis

**DOI:** 10.1080/07853890.2026.2624215

**Published:** 2026-02-23

**Authors:** Tianshu Ying, Shaolong E., Huanchun Ying

**Affiliations:** ^a^ Department of Oncology, Shengjing Hospital of China Medical University, Shenyang, China; ^b^ Department of Urology, Shengjing Hospital of China Medical University, Shenyang, China; ^c^ Department of Obstetrics and Gynecology, Shengjing Hospital of China Medical University, Shenyang, China

**Keywords:** Platinum-resistant/refractory ovarian cancer (PROC), molecular targeted drugs, bevacizumab, sorafenib, Bayesian network meta-analysis

## Abstract

**Background:**

Although single-agent chemotherapy is the most common approach for treating platinum-resistant or refractory ovarian cancer (PROC), there is growing evidence that combining molecular targeted agents with chemotherapy is beneficial, especially for certain patient groups. However, the most effective combination regimen remains elusive.

**Objectives:**

This Bayesian network meta-analysis (NMA) aims to identify the best combination therapy for PROC.

**Methods:**

Relevant studies were searched in PubMed, EMBASE, Web of Science and the Cochrane Central Register of Controlled Trials from their inception until October 2024. The primary outcomes were overall survival (OS), progression-free survival (PFS) and adverse events (AEs). Statistical analyses were performed using the GEMTC package (1.0-2) and R 4.2.0. This review was registered in PROSPERO (CRD42023428414).

**Results:**

Our analysis of 22 randomized controlled trials (RCTs) (*n* = 3408) demonstrated that chemotherapy combinations with bevacizumab (hazard ratio (HR) = 0.52–0.65), sorafenib (HR = 0.65, 95% confidence interval (CI): 0.45–0.93) or adavosertib (HR = 0.56, 95%CI: 0.35–0.90) significantly improved OS and PFS versus chemotherapy alone. Notably, adavosertib + gemcitabine was associated with an increased risk of grade 3–4 AEs (relative risk (RR) = 1.8, 95%CI: 1.3–2.7), but these were generally manageable.

**Conclusions:**

Bevacizumab-based combinations demonstrate consistent benefits across multiple regimens for PROC. Paclitaxel + bevacizumab emerges as the optimal balance of efficacy and safety. Topotecan + sorafenib could be an alternative for patients who are ineligible for anti-angiogenic therapy.

## Key Messages

- Targeted therapy + chemotherapy may be superior to standalone chemotherapy in PROC.
- Gemcitabine + bevacizumab can improve PFS of PROC patients.
- While effective, adavosertib + gemcitabine is associated with increased toxicity, requiring careful patient selection.

## 1. Introduction

Platinum-resistant/refractory ovarian cancer (PROC) represents a critical clinical challenge that develops in nearly 80% of patients initially diagnosed with platinum-sensitive ovarian cancer (OC) and who experience disease recurrence [1]. Platinum-refractory disease indicates progression during platinum therapy or within four weeks after the last dose. Platinum-resistant disease indicates progression within six months after completing therapy [2]. Among recurrent OC cases, approximately, 25–30% demonstrate platinum resistance at first relapse. This proportion increases substantially with subsequent lines of therapy [3]. PROC patients have a particularly dismal prognosis, with response rates to subsequent chemotherapy reported to be below 15% and median overall survival (OS) typically less than 12 months [4,5]. Even with current standard treatments, such as non-platinum single-agent chemotherapy ± bevacizumab, progression-free survival (PFS) is limited to three to four months [3]. This poor outlook is compounded by the heterogeneous nature of PROC, which arises from diverse molecular resistance mechanisms and is often diagnosed late due to vague symptoms and a lack of biomarkers [1,6,7]. The high recurrence rates and rapid development of cross-resistance to multiple agents underscore the urgent need for novel therapeutic strategies [8,9].

Non-platinum-based agents, such as liposomal doxorubicin, weekly paclitaxel with or without pazopanib, topotecan, gemcitabine and etoposide, are the preferred regimens for the treatment of PROC [10]. However, the response rate to sequential therapy with these individual agents remains low at 10–30% [11]. Genetic testing has expanded the treatment options for OC beyond simply chemotherapy [12]. Molecular targeted therapies are a type of treatment modality that targets and disrupts specific processes and signalling pathways critical for cancer cell growth, proliferation and survival. These therapies encompass several categories of drugs [13,14], including conventional small-molecule inhibitors and hormonal drugs [15]. Additionally, immune checkpoint inhibitors (ICIs), such as those targeting programmed cell death protein 1 (PD-1) [16] and cytotoxic T lymphocyte-associated protein 4 (CTLA-4) [17], are integral components of this therapeutic class. Furthermore, targeted cytotoxic therapies, exemplified by the dihydrofolate reductase inhibitor methotrexate [18], constitute another facet of this approach.

Several anti-angiogenic agents have been shown to be effective in patients with recurrent OC. These agents include bevacizumab (anti-vascular endothelial growth factor (anti-VEGF)), apatinib (anti-vascular endothelial growth factor receptor-2 (anti-VEGFR-2)), olaparib and niraparib (poly(ADP-ribose) polymerase-1 (PARP-1) and PARP-2 inhibitors) [11,19,20]. In addition to the scarcity of systematic reviews and meta-analyses investigating pharmacological interventions for PROC, the identification of the most effective therapeutic strategies has been hampered by limited research in PROC [21] and inadequate understanding of targeted drugs in combination with chemotherapeutics [22–25]. There is increasing evidence demonstrating the efficacy of anti-angiogenic therapy and chemotherapy in improving outcomes in PROC [26]. Mechanistically, anti-angiogenic therapy can normalize abnormal tumour vasculature and improve perfusion, thereby enhancing the delivery of cytotoxic drugs [27,28]. Interestingly, the prognosis of PROC patients treated with targeted drugs varies depending on the chemotherapeutic agent used in the combination therapy [29]. However, the mechanisms that contribute to the differences in efficacy among different combinations of targeted therapy and chemotherapy are currently unclear.

Due to heterogeneous study designs and a lack of direct comparisons, identifying the most effective therapeutic strategies remains challenging [30]. We conducted a network meta-analysis (NMA) to gain insights into the effectiveness of different combination therapies. NMA generates estimates of the relative effects of each therapy in comparison with all other therapies within a network, linked by direct and indirect comparisons derived from randomized controlled trial (RCT) data. This approach allows us to determine the probability that a specific therapy can result in a particular outcome, and rank the various treatment options for each outcome from most to least effective. Here, we evaluated the efficacy of current combinations of targeted therapy and chemotherapy in PROC patients using the NMA approach. Existing NMAs have provided preliminary evaluations of efficacy and safety [21]. Yet, with limited earlier evidence, adequate direct comparisons and comprehensive assessments were not feasible. Subgroup analyses exploring combinations of chemotherapy and targeted agents were also constrained. With more evidence now available, these gaps can be addressed, and the present NMA was therefore designed.

## 2. Methods

The present NMA was conducted in accordance with the NMA statement of the Preferred Reporting Items for Systematic Reviews and Meta-analyses (PRISMA2020) [31]. This study was prospectively registered with PROSPERO under the identification number CRD42023428414.

### 2.1. Literature search

Relevant articles were searched in PubMed, EMBASE, Web of Science and the Cochrane Central Register of Controlled Trials from inception to October 2024 using subject terms and free-text keywords without any language, publication date or regional restrictions. The search strategy is outlined in Supplementary Table S1.

### 2.2. Literature selection, eligibility criteria and data extraction

The identified articles were imported into reference management software to remove duplications. After screening the titles and abstracts, the full texts of potentially eligible articles were downloaded for further eligibility evaluation.

*Inclusion criteria*: (1) Patients diagnosed with OC who experienced recurrence within 6 months after platinum chemotherapy; (2) outcome measures encompassed OS, PFS, grade 3–4 adverse events (AEs) and phase II or III RCTs; (3) comparison of targeted drugs in combination with chemotherapy and chemotherapy alone; (4) RCTs. The comparisons included (i) targeted therapy plus chemotherapy versus chemotherapy alone and (ii) different targeted therapy plus chemotherapy regimens; (5) including both platinum-resistant (recurrence within 6 months) and platinum-refractory (progression during or within 4 weeks after the last platinum dose) disease.

*Exclusion criteria*: (1) One-arm studies, dose-finding studies, conference abstracts or systematic reviews/meta-analyses; (2) inaccessible full texts; (3) if multiple original studies investigated the same outcome measures based on the same RCT, only the study with the larger sample size was selected; and (4) trials in which the targeted agent was administered only as maintenance therapy after completing chemotherapy were excluded.

Data pertaining to the details of the RCTs were extracted into a standardized data extraction form, including: (1) first author’s name, publication year, trial number, sample sizes of both intervention groups, and total participants; (2) patient demographics including median age, FIGO stage, tumour grade, ECOG performance status, percent of patients with ascites and serous histology type, and percent of patients who received targeted therapy and chemotherapy regimens; and (3) outcome measures including OS, PFS and grades 3–4 AEs. Literature screening and data extraction were performed independently by two investigators (TSY, ESL), followed by a cross-checking procedure. Any disagreement was resolved by consulting a third investigator (LC).

### 2.3. Quality assessment

The quality of the included RCTs was assessed by two investigators (ESL, TSY) using the Cochrane Collaboration’s Risk of Bias tool for RCTs (RoB 1) [32]. This tool encompassed seven domains, namely random sequence generation, allocation concealment, blinding of participants and personnel, blinding of outcome assessors, incomplete outcome data, selective reporting and other sources of bias. Each domain was scored as having a low, high or unclear risk of bias. After quality assessment, the two investigators cross-checked their results. Dissents, if any, were settled by a third investigator (HCY) (Supplementary Table S2).

### 2.4. Statistical analysis

OS and PFS were synthesized utilizing hazard ratios (HRs) as effect sizes, whereas AEs were synthesized using risk ratios (RRs). The 95% credible intervals (CrIs) were computed for both measures. NMA was conducted using a Bayesian random-effects model constructed based on the Markov chain Monte Carlo method, which involved running four parallel Markov chains with an annealing time of 20,000 iterations. After completing 50,000 simulation iterations, the fit and global consistency of the model were evaluated using the deviance information criterion (DIC). In cases where a closed-loop network was identified, local consistency was examined by the node-splitting approach. Interventions were ranked based on the area under the cumulative ranking curve. Surface under the cumulative ranking curve and league tables was constructed to elucidate the differences in outcomes among various interventions. The overall heterogeneity statistic (*I*^2^) was calculated, and both fixed-effect and random-effects models were fitted. The DIC values of the two models were compared. If the overall heterogeneity was high (*I*^2^ > 50%) or if the difference in DIC exceeded 5, a random-effects model was used. All statistical analyses were performed in R 4.2.0 (R Development Core Team, Vienna, Austria, http://www.R-project.org), and the Bayesian NMA was constructed using the GEMTC package (1.0-2). Due to the heterogeneity of study populations and interventions, no pre-specified subgroup analyses were conducted. Following data extraction, pre-specified subgroup analyses were performed based on the chemotherapy backbone and the class or specific type of concurrent targeted agent within each treatment regimen.

## 3. Results

### 3.1. Literature search

**Figure 1.**
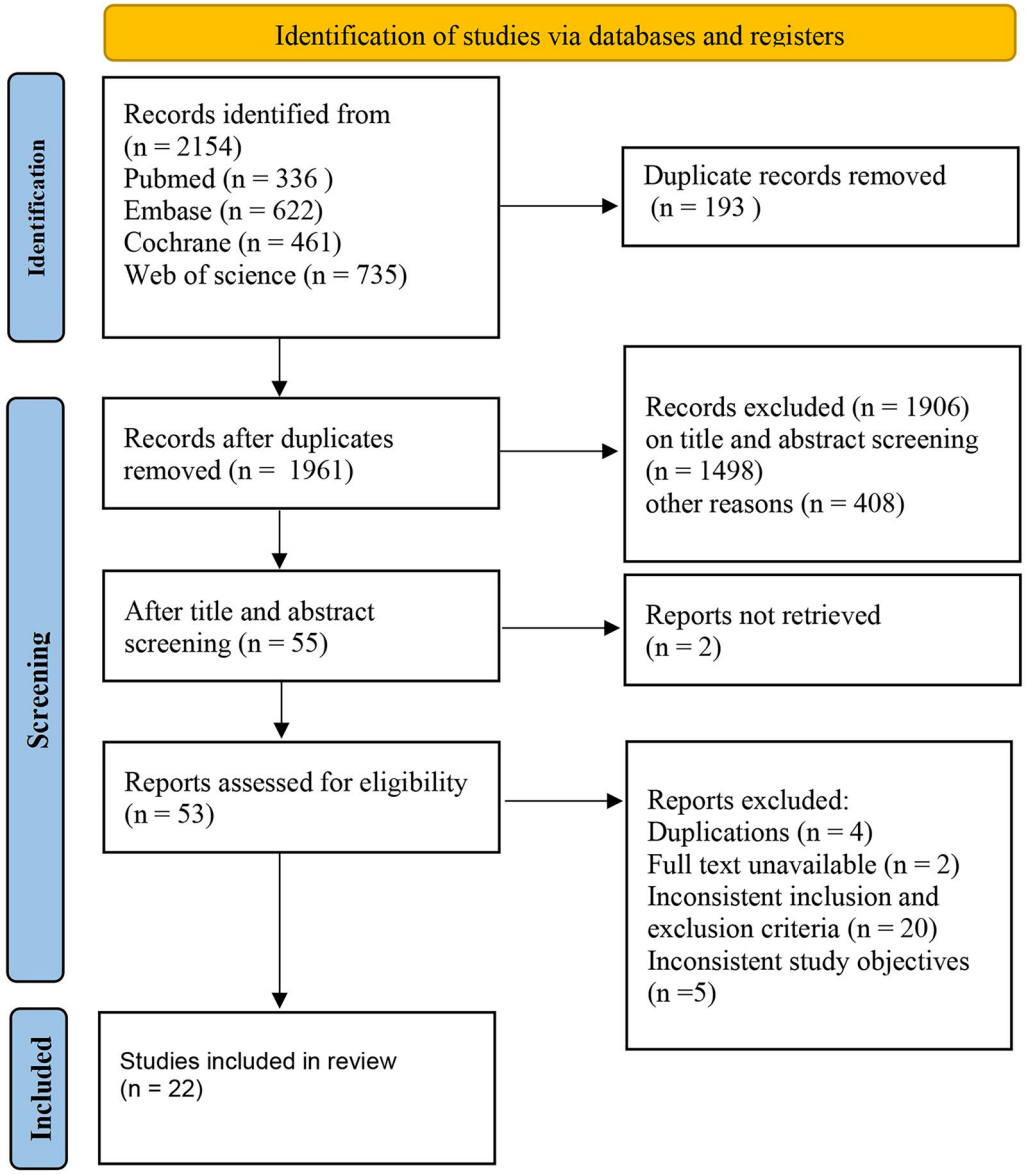
Flowchart of literature screening. Other reasons for exclusion include inconsistent interventions, populations, animal experiments, meta-analyses, conference abstracts, online publications, reviews, editorial material, retrospective studies and non-RCTs.

Of the 2154 studies initially identified (search updated in October 2024), 29 duplicates were removed and 2099 were excluded based on title and abstract screening. After full-text review, an additional 33 studies were excluded (detailed reasons for exclusion are provided in Supplementary Table S3). Finally, a total of 22 RCTs involving 3408 PROC patients were included in the NMA. A flowchart of the study selection process is shown in Figure 1. The baseline characteristics of the included studies, including sample size, study period, geographic regions and patient demographics, are summarized in Table 1. Detailed information on the chemotherapy regimens and targeted agents used across trials is provided in Supplementary Table S4, while the dosing regimens and line-of-therapy settings of non-standard investigational drugs are outlined in Supplementary Table S5.

**Table 1.** General characteristics of the included studies.

Study ID	NCT number	No. of pt.	Period	Regions	Age	ECOG	Serous OC (%)	Target treatment	Other treatment
Banerjee et al. [33]	ISRCTN16426935	140	201601–201803	UK	63.00, 7.80	NA	100%	Arm 1: mTOR (vistusertib) Arm 2: PLB	Arm 1: paclitaxel Arm 2: paclitaxel
Sharma et al. [34]	NA	75	201710–201909	India	53.10, 9.12	0/1/2: 1/29/45	85.33%	Arm 1: VEGFRi (pazopanib) Arm 2: none	Arm 1: EC^h^ Arm 2: EC^h^
Liu et al. [35]	NCT01447706	223	201202–201303	USA, Canada and Europe	60.26, 47.73	0–1/2: 213/10	70.85%	Arm 1: HER3 (seribantumab) Arm 2: none	Arm 1: paclitaxel Arm 2: paclitaxel
Lheureux et al.[36]	NCT02151292	94 (61/33)	201409–201805	USA and Canada	62.12, 2.86	0/1/2: 27/63/4	NA	Arm 1: WEE1 (adavosertib) Arm 2: PLB	Arm 1: GC Arm 2: GC
Pignata et al. [26]	NCT01644825	73	201012–201302	Italy	56.47, 9.30	NA	68.49%	Arm 1: VEGFR (pazopanib) Arm 2: none	Arm 1: paclitaxel Arm 2: paclitaxel
McNeish et al.[37]	NCT01196741	107	201104–201205	UK	62.47, 11.37	0/1/2: 38/65/4	73.83%	Arm 1: SRC (saracatinib) Arm 2: PLB	Arm 1: paclitaxel Arm 2: paclitaxel
Duska et al.[38]	NCT01610206	148	201208–201703	USA	NA	NA	NA	Arm 1: VEGFR (pazopanib) Arm 2: none	Arm 1: GC Arm 2: GC
Naumann et al.[39]	NCT00722592	149	200809–201006	USA, Canada and Poland	NA	NA	68.46%	Arm 1: FR (vintafolide) Arm 2: none	Arm 1: PLD Arm 2: PLD
Pujade-Lauraine et al. [40]	NCT02580058	566 (188/190/188)	201601–201705	23 regions^e^	60.36, 2.90	0/1/≥2: 286/277/2	73.50%	Arm 1: anti-PD-L1 (avelumab) Arm 2: none Arm 3: anti-PD-L1 (avelumab)	Arm 1: PLD Arm 2: PLD Arm 3: none
Konstantinopoulos et al. [41]	NCT02595892	70(34/36)	201702–201809	USA	NA	0/1: −42/28	NA	Arm 1: ART (berzosertib) Arm 2: none	Arm 1: GC Arm 2: GC
Pujade-Lauraine et al. [19]	NCT00976911	361	200910–201104	France	60.89, 10.55	0/1/2/missing: 206/127/23/5	73.41%	Arm 1: VEGFi (bevacizumab) Arm 2: none	Arm 1: mixed^c^ Arm 2: Mixed^c^
Lee et al. [42]	NCT03699449	23 (18/5)	201812–202010	Korea	55.48, 9.00	0/1: 4/19	NA	Arm 4: anti-CTLA-4 (tremelimumab) Arm 3: none	Arm 4: mixed^b^ Arm 3: mixed^b^
Makhija et al.[43]	NCT00096993	130	200502–200608	USA	58.17, 13.90	0/1–2: 85/45	NA	Arm 1: HER2i (pertuzumab) Arm 2: PLB	Arm 1: GC Arm 2: GC
Kurzeder et al. [29]	NCT01684878	156	201310–201409	10 regions^f^	63.15: 10.49	0/1/2/3: 79/62/14/1	76.92%	Arm 1: HER2 (pertuzumab) Arm 2: PLB	Arm 1: mixed Arm 2: mixed^a^
Shoji et al.[44]	JGOG3023	103	201506–201911	Japan	60.50, 10.93	0/1/2/3/4/missing: 86/15/0/0/0/2	62.14%	Arm 1: VEGFi (bevacizumab) Arm 2: none	Arm 1: mixed^d^ Arm 2: mixed^d^
Liu et al.[45]	NA	86	201403–201603	China	47.04, 8.57	0/1: 46/40	55.81%	Arm 1: VEGF (bevacizumab) Arm 2: none	Arm 1: paclitaxel Arm 2: paclitaxel
Oza et al.[46]	NCT00889382	152 (51/51/50)	2009–2014	NA	56.89, 10.17	0/1/2: 85/66/1	NA	Arm 1: IGF-1 (linsitinib) intermittent Arm 2: IGF-1 (linsitinib) continuous Arm 3: none	Arm 1: paclitaxel Arm 2: paclitaxel Arm 3: paclitaxel
Roque et al. [47]	NCT3093155	76	201703–202011	USA	66.37, 8.89	0–1/2: 176/24	82.89%	Arm 1: VEGF (bevacizumab) Arm 2: none	Arm 1: IXA Arm 2: IXA
McGuire et al. [48]	NCT00913835	163	200906–201402	USA, UK and Spain	59.30, 9.860	0/1: 68/55	NA	Arm 1: PDGFRα (olaratumab) Arm 2: none	Arm 1: PLD Arm 2: PLD
Chekerov et al. [49]	NCT01047891	174	201001–201309	Germany	57.83, 10.36	0/1/2/NA: 92/71/4/5	79.07%	Arm 1: RAF + VEGFR + PDGFR (sorafenib) Arm 2: PLB	Arm 1: topotecan Arm 2: topotecan
Marth et al.[50]	NCT01281254	223 (114/109)	201111–201301	16 regions^g^	60.16, 10.55	0/1/2: 142/80/1	76.68%	Arm 1: Ang1 + Ang2 + Tie2 (trebananib) Arm 2: PLB	Arm 1: PLD Arm 2: PLD
Joly et al. [51]	NCT02383251	116	201506–201904	France	65.14, 8.77	0/1/2: 46/28/1	88.79%	Arm 1: VEGFR (pazopanib) Arm 2: none	Arm 1: paclitaxel Arm 2: paclitaxel

PLD: doxorubicin; NA: not applicable; JGOG: Japanese Gynecological Oncology Group; NCT: ClinicalTrials.gov Identifier; no. of pt.: number of patients; ECOG: Eastern Cooperative Oncology Group Performance Status; PLB: placebo; int: intermittent; con: continuous; GC: gemcitabine; IXA: ixabepilone.

^a^ Topotecan, paclitaxel or gemcitabine.

^b^ PLD or topotecan or paclitaxel or durvalumab.

^c^ Paclitaxel and doxorubicin or topotecan.

^d^ Doxorubicin or topotecan or paclitaxel or gemcitabine.

^e^ Australia, Austria, Belgium, Canada, Czech Republic, Denmark, France, Greece, Hong Kong, Hungary, Ireland, Israel, Japan, the Netherlands, Norway, Poland, Russia, Singapore, South Korea, Spain, Switzerland, Taiwan, the UK and the USA.

^f^ Spain, Germany, Belgium, France, Italy, Netherlands, Norway, Sweden, Denmark and Austria.

^g^ Austria, Belgium, Canada, Denmark, Finland, France, Germany, Italy, Japan, Netherlands, New Zealand, Norway, Spain, Sweden, UK and USA.

^h^ Etoposide + cyclophosphamide.

### 3.2. Quality assessment

The risk of bias in the included RCTs was generally low. Random sequence generation and allocation concealment were scored as medium or low risk of bias in 19 (86.36%) RCTs as the authors provided detailed descriptions of the randomization methods employed. However, three open-label RCTs (13.64%) had a high risk of bias in blinding of participants and personnel. Blinding of outcome assessments, incomplete data and selective reporting were all rated as low-risk in all included RCTs. Quality assessment results are summarized in Table 2. For the domains judged as high risk, we summarized these items further and provided the detailed reasons for the judgments in Supplementary Table S6.

**Table 2.** Risk of bias assessment for the included studies.

Study	#1	#2	#3	#4	#5	#6	#7	Overall quality
Roque et al. [47]	Low	Low	Unclear	Unclear	Low	Unclear	Unclear	High
Banerjee et al. [33]	Low	Low	Low	Unclear	Low	Low	Unclear	High
Chekerov et al. [49]	Low	Low	Low	Unclear	Low	Low	Low	High
Duska et al. [38]	Low	Unclear	High	Low	Low	Unclear	Unclear	Medium
Joly et al. [51]	Low	Unclear	High	Unclear	Low	Low	Unclear	Medium
Konstantinopoulos et al. [41]	Low	Unclear	High	Unclear	Low	Unclear	Low	Medium
Kurzeder et al. [29]	Unclear	Low	Low	Low	Unclear	Unclear	Low	High
Lee et al. [42]	Low	High	High	Low	Low	Unclear	Unclear	Low
Lheureux et al. [36]	Low	Low	Low	Unclear	Low	Low	Low	High
Liu et al. [35]	Unclear	Unclear	High	Unclear	Unclear	Low	Unclear	Medium
Liu et al. [45]	Low	Unclear	Unclear	Unclear	Unclear	Unclear	Unclear	Medium
Makhija et al. [43]	Unclear	Low	Low	Unclear	Low	Low	Unclear	High
Marth et al. [50]	Unclear	Unclear	Low	Unclear	Low	Low	Unclear	High
McGuire et al. [48]	Low	Unclear	High	Unclear	Unclear	Unclear	Unclear	Medium
McNeish et al. [37]	Unclear	Low	Low	Unclear	Low	Unclear	Unclear	High
Naumann et al. [39]	Unclear	Unclear	Unclear	Low	Low	Unclear	Unclear	Medium
Oza et al. [46]	Unclear	High	High	Unclear	Low	Low	Low	Low
Pignata et al. [26]	Unclear	Unclear	High	Unclear	Low	Low	Unclear	Medium
Pujade-Lauraine et al. [19]	Unclear	Unclear	High	Unclear	Low	Unclear	Low	Medium
Pujade-Lauraine et al. [40]	Low	High	High	Low	Low	Unclear	Unclear	Low
Sharma et al. [34]	Low	Unclear	High	Unclear	Unclear	Low	Unclear	Medium
Shoji et al. [44]	Low	Unclear	High	Unclear	Low	Low	Low	Medium

#1: random sequence generation; #2: allocation concealment; #3: blinding of subjects and personnel; #4: blinding of outcome assessment; #5: incomplete data; #6: selective reporting; #7: other sources of bias.

### 3.3. General characteristics of included studies

**Figure 2.**
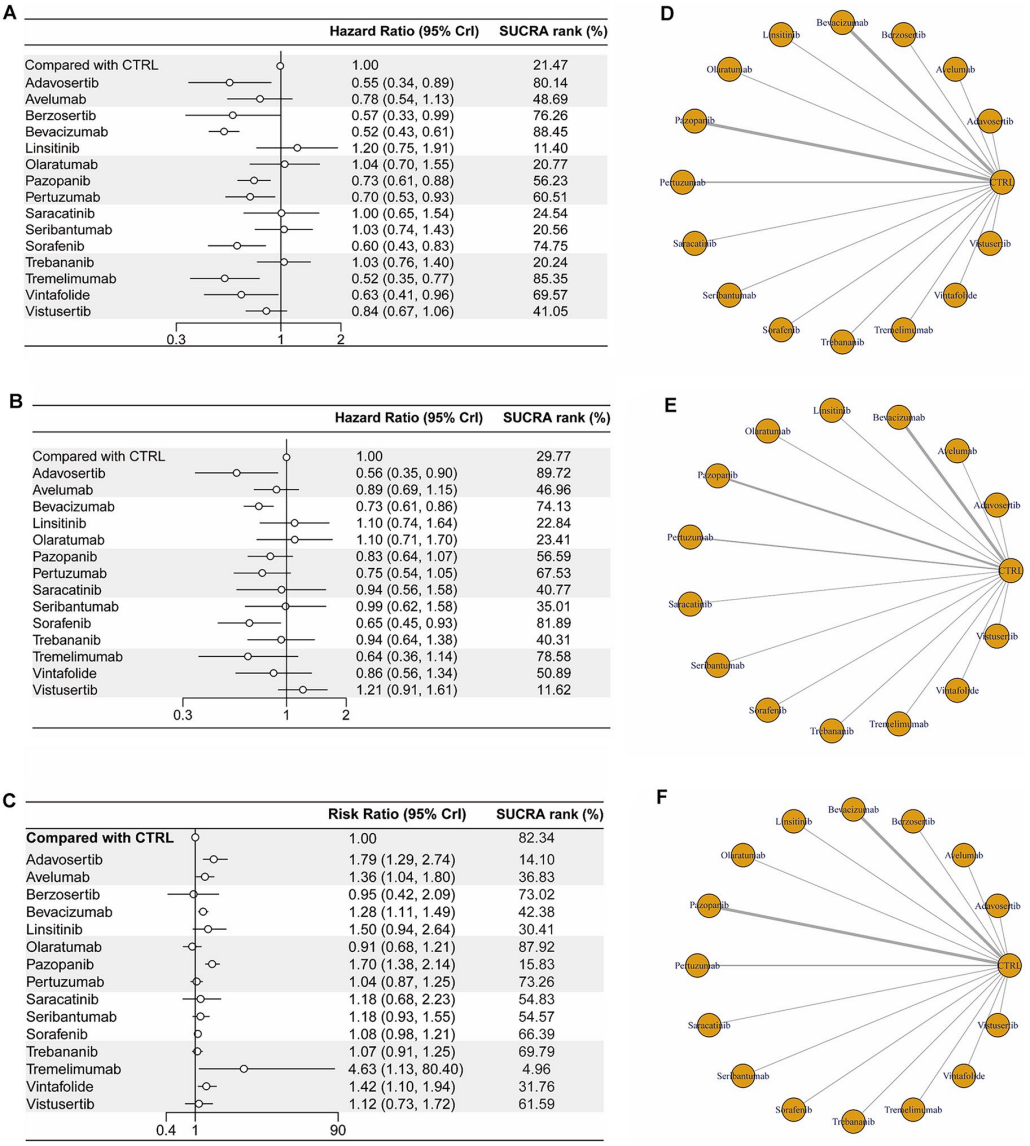
Forest plot and ranking for (A) PFS, (B) OS, (C) AEs, and the network diagram of the relationship among different interventions and their impact on (D) PFS, (E) OS and (F) AEs. The thickness of the lines connecting the interventions corresponds to the number of studies that directly compared them, with thicker lines indicating a greater number of studies.

The two types of interventions reported in the included studies were chemotherapy (ixabepilone, paclitaxel, topotecan, gemcitabine, doxorubicin and etoposide + cyclophosphamide) and targeted therapy (vistusertib, sorafenib, pazopanib, berzosertib, adavosertib, trebananib, saracatinib, vintafolide, linsitinib, bevacizumab, pertuzumab, tremelimumab, seribantumab, olaratumab and avelumab). The network for analysing all treatment regimens is shown in Figure 2 (PFS, OS and safety outcomes). The included studies ranged from 2005 to 2024, predominantly focusing on the period between 2010 and 2018. These studies were carried out across various countries and regions, encompassing North America (USA, Canada), Europe (UK, Spain, Italy, Germany, Belgium, France, Netherlands, Norway, Sweden, Denmark, Austria, Poland, Czech Republic), Asia (India, China, Japan, South Korea, Taiwan, Hong Kong) and Oceania (Australia, New Zealand). The participants in the included studies had predominantly serous OC, a median age of approximately 60 years, and a disease that minimally affected activities of daily living (Table 1).

### 3.4. NMA results

#### 3.4.1. Progression-free survival

Bevacizumab + chemotherapy (HR = 0.52, 95% confidence interval (CI): 0.43–0.61) and tremelimumab + chemotherapy (HR = 0.52, 95%CI: 0.35–0.77) resulted in longer PFS than chemotherapy alone (Figure 2(A): PFS). In addition, the Bayesian NMA revealed that pertuzumab (HR = 0.70, 95%CI: 0.53–0.93), pazopanib (HR = 0.73, 95%CI: 0.61–0.88), vintafolide (HR = 0.63, 95%CI: 0.41–0.96), sorafenib (HR = 0.60, 95%CI: 0.43–0.83), adavosertib (HR = 0.55, 95%CI: 0.34–0.89) and berzosertib (HR = 0.57, 95%CI: 0.33–0.99) significantly improved PFS compared to chemotherapy alone (Figure 2(A): PFS).

#### 3.4.2. Overall survival

Next, we compared 14 individual treatment nodes, including six immunotherapy nodes (olaratumab, bevacizumab, pertuzumab, avelumab, seribantumab and tremelimumab) and eight targeted drugs (vintafolide, olaratumab, bevacizumab, pertuzumab, avelumab, seribantumab, tremelimumab, sorafenib, linsitinib, vistusertib, pazopanib, saracatinib, adavosertib and trebananib). Our analysis showed that 175 mg of adavosertib + gemcitabine (HR = 0.56, 95%CI: 0.35–0.90) significantly improved the OS of PROC patients, followed by 400 mg of sorafenib + topotecan (HR = 0.65, 95%CI: 0.45–0.93) and bevacizumab + any chemotherapy (HR = 0.73, 95%CI: 0.61–0.86). The league table depicting the relative effects of all treatment pairs on OS is shown in Figure 2(B): OS and Supplementary Table S2.

#### 3.4.3. Adverse events

In terms of safety, grade 3–4 AEs were more common with tremelimumab (RR = 4.6, 95%CI: 1.1–80), adavosertib (RR = 1.8, 95%CI: 1.3–2.7), avelumab (RR = 1.4, 95%CI: 1.0–1.8), bevacizumab (RR = 1.3, 95%CI: 1.1–1.5), pazopanib (RR = 1.7, 95%CI: 1.4–2.1) and vintafolide (RR = 1.4, 95%CI: 1.1–1.9), but less common with berzosertib (RR = 0.95, 95%CI: 0.42–2.1), linsitinib (RR = 1.5, 95%CI: 0.94–2.6), olaratumab (RR = 0.91, 95%CI: 0.68–1.2), pertuzumab (RR = 1.0, 95%CI: 0.87–1.3), saracatinib (RR = 1.2, 95%CI: 0.68–2.2), seribantumab (RR = 1.2, 95%CI: 0.93–1.5), sorafenib (RR = 1.1, 95%CI: 0.98–1.2), trebananib (RR = 1.1, 95%CI: 0.91–1.2) and vistusertib (RR = 1.1, 95%CI: 0.73–1.7) (Figure 2(C): AEs). Across trials, the main grade 3–4 AEs were hematologic toxicities, hypertension, gastrointestinal events and dermatologic reactions. We validated these findings with a Bayesian NMA and found that sorafenib + topotecan significantly improved OS without increasing the incidence of AEs. We further categorized and summarized the specific types and frequencies of grade 3–4 AEs across the included trials. Detailed information is provided in Supplementary Table S7.

### 3.5. Subgroup analysis

**Figure 3.**
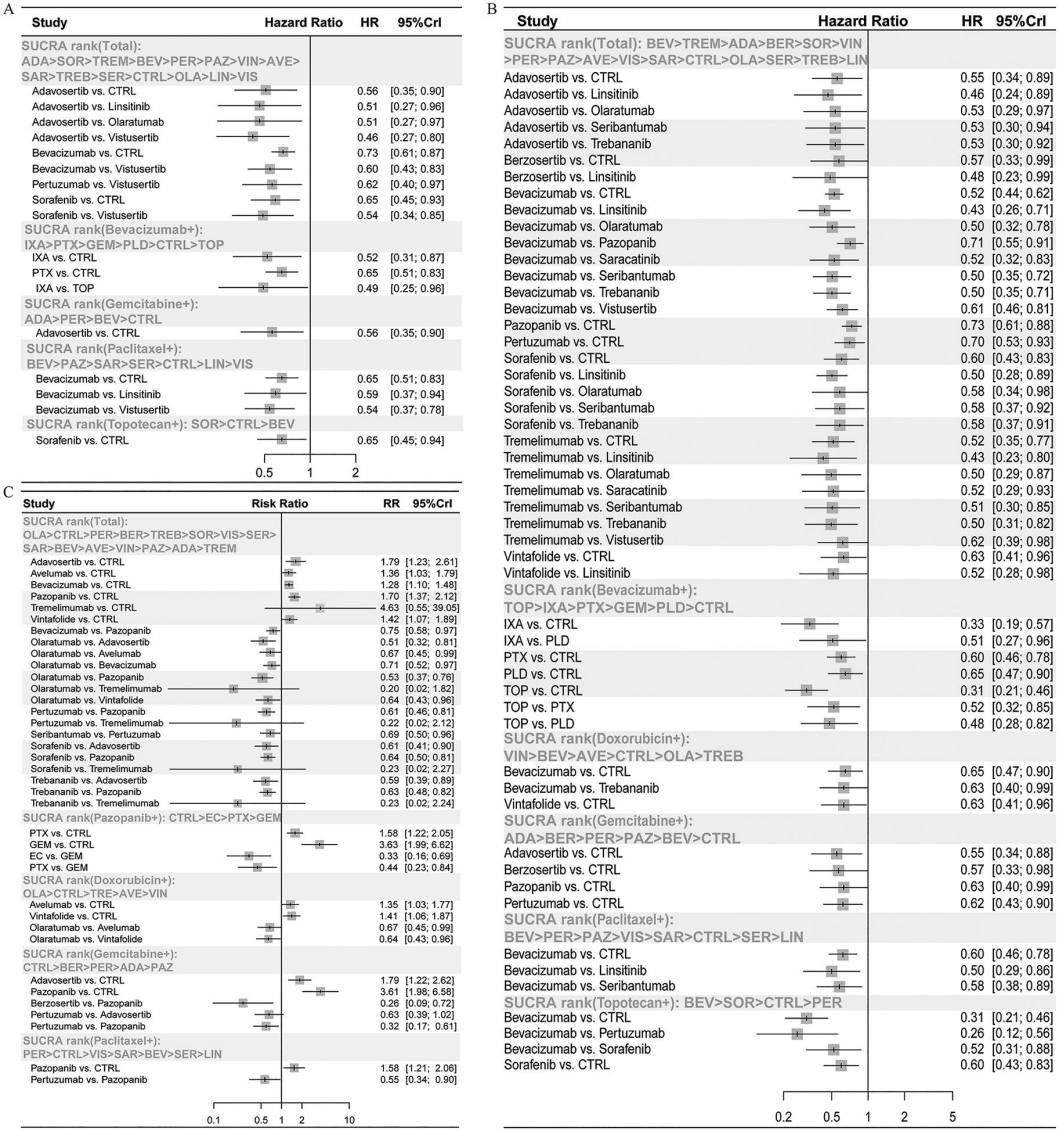
SUCRA ranking of all and subgroups of combination regimens for (A) OS, (B) PFS and (C) AEs. (+) In combination with ADA: adavosertib; SOR: sorafenib; TREM: tremelimumab; BEV: bevacizumab; PER: pertuzumab; PAZ: pazopanib; VIN: vintafolide; AVE: avelumab; SAR: saracatinib; TREB: trebananib; SER: seribantumab; CTRL: control; OLA: olaratumab; LIN: linsitinib; VIS: vistusertib; IXA: ixabepilone; PTX: paclitaxel; GEM: gemcitabine; PLD: doxorubicin; TOP: topotecan; BER: berzosertib; EC: etoposide + cyclophosphamide.

Subgroup analysis based on backbone chemotherapies revealed distinct efficacy and safety profiles across different combination regimens (Figure 3). Notably, paclitaxel plus bevacizumab improved both PFS and OS. In contrast, while doxorubicin- or gemcitabine-based combinations (e.g. combined with bevacizumab, adavosertib or pazopanib) improved PFS, their benefits on OS were less consistent or accompanied by increased toxicity. For instance, gemcitabine plus adavosertib improved OS but had a less favourable safety profile. Among topotecan-based regimens, the addition of sorafenib improved PFS and OS, with a higher incidence of AEs.

A reverse subgroup analysis by targeted agents was performed to provide complementary insights. Bevacizumab showed varying efficacy depending on its chemotherapeutic partner. It provided the most consistent PFS and OS benefits when combined with paclitaxel or ixabepilone. Pazopanib + etoposide + cyclophosphamide achieved competitive PFS with a relatively better safety profile compared to its combinations with other chemotherapeutic agents.

### 3.6. Inconsistency test and heterogeneity analysis

The majority of comparisons demonstrated a high level of clinical and methodological consistency across studies, as well as strong study agreement. However, since there is no ‘loop’ in the network, it is impossible to conduct an inconsistency test using the ‘nodesplit’ method.

## 4. Discussion

In this Bayesian NMA of 22 RCTs involving 3408 patients with platinum-resistant or refractory ovarian cancer (PROC), we identified several combination regimens that improved survival outcomes more consistently than chemotherapy alone. The most robust benefits in prolonging both OS and PFS were shown by adavosertib plus gemcitabine, bevacizumab-based combinations, and sorafenib plus topotecan. Notably, paclitaxel + bevacizumab and topotecan + sorafenib emerged as regimens balancing efficacy with an acceptable safety profile. For patients unable to tolerate these options, combinations such as gemcitabine + berzosertib or pertuzumab provided moderate PFS benefits with relatively mild toxicity. Furthermore, subgroup analyses revealed that the choice of chemotherapy backbone influenced outcomes. Bevacizumab combined with paclitaxel or ixabepilone produced the most consistent gains in OS and PFS. Pazopanib + etoposide + cyclophosphamide offered a potential alternative for patients in poorer health. Collectively, these findings suggest that tailoring targeted agent–chemotherapy combinations according to patient condition and tolerability is a better strategy than relying on chemotherapy alone.

Previous studies have shown that the application of specific targeted agents along with chemotherapy improves the outcomes of PROC patients. For example, topotecan + sorafenib can inhibit tumour neovascularization by targeting HIF-1α and VEGFRs, respectively, thereby impeding cancer cell access to essential nutrients [52,53]. In addition, sorafenib disrupts various signalling pathways responsible for tumour cell proliferation and survival, while topotecan induces G0/G1 and S phase arrest and apoptosis by inhibiting topoisomerase I, an enzyme important for DNA replication [54]. The synergistic interplay among these pharmaceutical agents enables them to target tumour cells via multiple mechanistic pathways, thereby potentiating their overall antineoplastic effect [55]. The antiangiogenic effect of sorafenib can improve intra-tumoural blood perfusion, consequently facilitating the infiltration of chemotherapeutic agents like topotecan into the central region of the tumour. This synergistic interaction enhances uniform drug distribution and overall therapeutic efficacy within the neoplasm [49].

Bevacizumab, paclitaxel and ixabepilone have been reported to exhibit synergistic interactions [47,56,57]. Bevacizumab deprives the tumour of nutrients and causes hypoxia in the tumour tissue, which renders tumour cells more sensitive to ixabepilone. Hypoxia can promote entry into the sub-G1 phase where ixabepilone, a microtubule destabilizing agent, exerts its maximal effect. Bevacizumab directly inhibits VEGF-mediated angiogenesis, whereas ixabepilone indirectly affects the tumour vasculature by disrupting microtubule dynamics and impairing microtubule-dependent intracellular transport, thus inhibiting tumour cell proliferation and suppressing the generation of new blood vessels. Given its capacity to diminish vascular permeability within the neoplasm, bevacizumab enhances the penetration and accumulation of paclitaxel and/or ixabepilone within the tumour microenvironment [58], augmenting their cytotoxic effects on the tumour [42,59]. Consequently, the synergistic antineoplastic activities of bevacizumab, paclitaxel and ixabepilone can improve the prognosis of PROC. Adavosertib is a Wee1 kinase inhibitor that promotes the entry and retention of tumour cells in the M phase, leading to accumulation of DNA damage and cell death [60–62]. The concurrent administration of adavosertib and gemcitabine has been shown to increase DNA damage through synergistic chemosensitization, thereby potentiating the cytotoxic effects of these therapeutic agents. Adavosertib increases the cytotoxic effect of gemcitabine on tumour cells by inhibiting Wee1, allowing biliary tract cancer cells to proliferate and continue to accumulate DNA damage [63]. In addition, adavosertib inhibits homologous recombination (HR) repair in locally advanced pancreatic cancer by suppressing the Wee1-mediated augmentation of gemcitabine sensitivity [64].

While the majority of combination regimens demonstrated a less favourable safety profile in comparison with monotherapies, several combination regimens exhibit an advantageous safety profile. Both targeted and chemotherapeutic drugs possess distinct toxicity profiles, and their combined use may result in a cumulative increase in toxicities, potentially elevating the incidence of adverse effects. For instance, targeted drugs may induce skin reactions, hypertension and gastrointestinal disturbances, whereas chemotherapeutic drugs commonly contribute to myelosuppression, nausea, alopecia and neurotoxicity [65,66]. Although adavosertib plus gemcitabine can prolong both the PFS and OS of PROC patients, this regimen was associated with a significant number of adverse effects. Gemcitabine is inherently myelosuppressive, leading to hematologic toxicities such as leukopenia, erythrocytosis and thrombocytopenia. The addition of adavosertib may exacerbate this myelosuppressive effect by disrupting cell cycle progression, increasing the likelihood of anaemia, infections and bleeding tendencies [67,68]. In contrast, the primary toxicity of topotecan is myelosuppression, manifesting as leukopenia, anaemia and thrombocytopenia, whereas sorafenib typically causes dermatological reactions on the hands and feet, hypertension, fatigue and diarrhoea [69,70]. Given their distinct mechanisms of toxicity, these drugs may result in less pronounced cumulative toxicities when used in combination. It has been posited that sorafenib possibly reduces tumour resistance to topotecan through the suppression of angiogenic and proliferative signalling pathways, while topotecan enhances the antitumour effects of sorafenib by directly inducing DNA damage. In clinical practice, efficacy must be balanced against toxicity. Adavosertib + gemcitabine demonstrated the greatest OS and PFS gains, but significant haematologic toxicity limits its use to fit patients only. In contrast, paclitaxel + bevacizumab and sorafenib + topotecan achieved survival benefits with manageable toxicity, making them preferable for broader populations. Bevacizumab-based regimens, meanwhile, remain suitable for frail patients. Overall, bevacizumab + paclitaxel and sorafenib + topotecan maintained a favourable balance between efficacy and safety. The toxicities associated with these regimens were generally manageable and tolerable. In contrast, adavosertib + gemcitabine and pazopanib-based regimens were associated with more severe adverse effects. Their toxicities were more limiting and potentially restricted broader clinical application.

This NMA extends previous findings by comprehensively comparing a broad spectrum of targeted-agent combinations for PROC to evaluate their efficacy and safety. Prior meta-analyses and the AURELIA trial have established the role of bevacizumab-based regimens in significantly improving PFS and OS, as indicated in current clinical guidelines [19,21,22,71]. Unlike earlier investigations, our NMA encompasses a substantially larger cohort of studies, performs detailed subgroup analyses for each agent, and directly evaluates the comparative effectiveness of various targeted agents [21,72]. This approach allows us to identify not only the most efficacious regimens but also those with the most favourable efficacy–safety balance.

Several limitations must be considered in this study. First, we only examined combinations of targeted drugs and chemotherapeutic agents but did not include additional single-agent or drug combination regimens. Second, the small sample size for certain combination treatment regimens may introduce bias into the study results. Third, among the populations covered in the included studies, there were more participants from Europe and the USA than from other countries, which may confound the interpretation of results. Fourth, we incorporated 22 RCTs, among which only three studies reported data on BRCA mutation status. Despite the well-established significance of BRCA mutations in OC, the limited data availability hinders our thorough evaluation of their role in treating platinum-resistant recurrent OC. Fifth, heterogeneity in prior lines of therapy across the included RCTs, variability in PFS and OS definitions, and incomplete reporting of BRCA/HRD status may affect the consistency of results and limit biomarker-based interpretation. Lastly, the absence of closed loops precluded the assessment of inconsistencies. Our recommendations for bevacizumab-based regimens should be cautious in patients with prior exposure to bevacizumab. Given a lack of baseline data, our findings may primarily apply to bevacizumab-naive patients, as prior use could reduce treatment response due to acquired resistance. Future practice and trials should document or stratify participants by prior bevacizumab exposure to clarify efficacy in this subgroup. Therefore, future studies should also prioritize biomarker-driven approaches (e.g. BRCA, HRD and angiogenic signatures) to refine patient selection, while evaluating cost-effectiveness in low-resource settings. Notably, while mechanistically supported, direct evaluation of PARPi and chemotherapy combinations in RCTs is limited. Ongoing trials combining PARP inhibitors with anti-angiogenic agents or ICIs may provide promising evidence to expand therapeutic options for PROC.

## 5. Conclusions

Based on our NMA, paclitaxel plus bevacizumab is the optimal balance of efficacy and tolerability for PROC. Bevacizumab-based combinations demonstrate consistent benefits across multiple regimens. For patients who are ineligible for anti-angiogenic therapy, topotecan–sorafenib is an alternative option. These findings support current guideline recommendations and highlight the need for biomarker-driven selection of targeted agents.

## Supplementary Material

Supplementary Table S5.docx

Supplementary Table S1.docx

Supplementary Table S4.docx

Supplementary Table S7.docx

Supplementary Table S2.docx

Supplementary Table S6.docx

Supplementary Table S3.docx

## Data Availability

The data generated in this study are available upon request from the corresponding author.
