# Supplementary material for: Molecular targeted therapy in combination with chemotherapy for the treatment of platinum-resistant/refractory ovarian cancer (PROC): a systematic review and network meta-analysis
Source: Ann Med. 2026 Feb 23;58(1):2624215. doi: 10.1080/07853890.2026.2624215 (PMC12931348; doi:10.1080/07853890.2026.2624215)
Supplement: Supplementary Table S5.docx [file IANN_A_2624215_SM0126.docx]

**Supplementary Table S5.** Details of non-standard PROC drugs included in this study.

| Study | Medication of non-standard PROC drugs | Status | Line of therapy | Dosing |
| --- | --- | --- | --- | --- |
| Banerjee (2022) | Vistusertib | Investigational | ≥ 2nd line PROC | Paclitaxel 80 mg/m² IV D1, 8, 15 plus Vistusertib 50 mg bd D1–3, 8–10, 15–17 of a 28d cycle |
| Sharma (2021) | Pazopanib | Investigational | 1 – 4 prior lines ( ≥ 2nd line PROC) | Arm A: Etoposide 50 mg D1–14 + Cyclophosphamide 50 mg D1–28 q4w; Arm B: same + Pazopanib 400 mg OD |
| Liu (2016) | Seribantumab | Investigational | ≥ 2nd line PROC | Seribantumab 40 mg/kg IV loading dose, then 20 mg/kg IV once per week; Paclitaxel 80 mg/m² IV weekly for 3 weeks followed by 1 week of rest |
| Lheureux (2021) | Adavosertib | Investigational | Median 3 prior regimens (range 1 – 5) | Gemcitabine 1000 mg/m² IV on days 1, 8, 15 + Adavosertib 175 mg PO once daily on days 1, 2, 8, 9, 15, 16, q28d cycle |
| Pignata (2016) | Pazopanib | Investigational | ≤ 2 prior chemotherapy lines (platinum-resistant/refractory) | Paclitaxel 80 mg/m² IV D1, 8, 15 q28d ± Pazopanib 800 mg PO daily, continuously |
| McNeish (2014) | Saracatinib | Investigational | Median 2 prior lines (range 1 – 7) | Saracatinib 175 mg o.d. or matched placebo 7 days before weekly paclitaxel (wPxl); wPxl: 80 mg/m² IV on days 1, 8, 15, 22, 29, 36 of an 8-week cycle, followed by 2-week break |
| Duska (2019) | **Pazopanib** | Investigational | 1 – 3 prior lines ( ≥ 2nd line PROC, including platinum-resistant and platinum-sensitive patients) | Gemcitabine 1000 mg/m² on days 1 and 8 of a 21-day cycle ± Pazopanib 800 mg orally daily |
| Naumann (2013) | Vintafolide | Investigational | Patients with recurrent platinum-resistant ovarian cancer, required ≥ two prior systemic cytotoxic regimens | PLD 50 mg/m² IV day 1 q28d ± Vintafolide 2.5 mg IV three times per week during weeks 1 and 3 of each 28-day cycle |
| Pujade-Lauraine (2021) | Avelumab | Investigational | Platinum-resistant: no prior systemic therapy; Platinum-sensitive: ≤ 3 prior lines (most recent containing platinum) | Avelumab 10 mg/kg IV q2w; PLD 40 mg/m² IV q4w |
| Konstantinopoulos (2020) | Berzosertib | Investigational | Patients with recurrent platinum-resistant high-grade serous ovarian cancer; prior 1 – 3 lines of systemic therapy | Gemcitabine 1000 mg/m² IV D1, 8 q21d plus Berzosertib 210 mg/m² IV D2, 9 q21d |
| Lee (2022) | Olaparib + Cediranib | Investigational | HRD-positive PROC; ≥ 2 prior lines | Olaparib 200 mg po bid + Cediranib 30 mg po qd |
|  | Olaparib + Durvalumab |  |  | Olaparib 300 mg po bid + Durvalumab 1,500 mg IV q4w |
|  | Durvalumab + CT (PLD or Topotecan or Weekly Paclitaxel) |  |  | Durvalumab 1,500 mg IV q4w + PLD 40 mg/m² IV q4w or Topotecan 4 mg/m² IV D1, 8, 15 q4w or Paclitaxel 80 mg/m² IV D1, 8, 15, 22 q4w (6 cycles) |
|  | Durvalumab + Tremelimumab (75 mg) + CT |  |  | Durvalumab 1,500 mg IV q4w + Tremelimumab 75 mg IV q4w (4 doses) + PLD 40 mg/m² IV q4w or Topotecan 4 mg/m² IV D1, 8, 15 q4w or Paclitaxel 80 mg/m² IV D1, 8, 15, 22 q4w (4 cycles) |
|  | Durvalumab + Tremelimumab (300 mg) + Weekly Paclitaxel |  |  | Durvalumab 1,500 mg IV q4w + Tremelimumab 300 mg IV (1 dose) + Paclitaxel 60 mg/m² IV D1, 8, 15 q4w (4 cycles) |
| Makhija (2010) | Pertuzumab + Gemcitabine | Investigational | Platinum-resistant; ≤ 1 prior regimen for resistant disease (any number for sensitive disease) | Avelumab 10 mg/kg IV every 2 weeks ± PLD 40 mg/m² IV every 4 weeks |
| Kurzeder (2016) | Pertuzumab | Investigational | Platinum-resistant ovarian cancer with low tumor HER3 mRNA expression; ≤ 2 prior lines of chemotherapy | Topotecan 1.25 mg/m² IV D1–5 q3w OR Paclitaxel 80 mg/m² IV D1, 8, 15 q3w OR Gemcitabine 1000 mg/m² IV D1, 8 q3w, plus Pertuzumab 840 mg IV loading, then 420 mg IV q3w |
| Oza (2018) | Linsitinib | Investigational | Recurrent platinum-resistant ovarian cancer; patients had received 1 – 3 prior chemotherapy regimens | Paclitaxel 80 mg/m² IV D1, 8, 15 q21d + Intermittent Linsitinib 600 mg po qd D1–3 weekly q21d (Arm A); Paclitaxel 80 mg/m² IV D1, 8, 15 q21d + Continuous Linsitinib 150 mg po bid continuously (Arm B) |
| McGuire (2018) | Olaratumab | Investigational | Platinum-refractory or resistant; 1 – 3 prior platinum regimens | Olaratumab 20 mg/kg IV D1, 15 q28d + Liposomal doxorubicin 40 mg/m² IV D1 q28d; crossover to Olaratumab monotherapy 20 mg/kg IV q2w upon progression |
| Chekerov (2018) | Sorafenib | Investigational | Platinum-refractory or resistant ovarian cancer; ≤ 2 prior chemotherapy lines for recurrent disease | Topotecan 1.25 mg/m² IV D1–5 q21d + Sorafenib 400 mg po bid D6–15 q21d (up to 6 cycles); responders could continue Sorafenib 400 mg po bid as maintenance |
| Marth (2017) | Trebananib | Investigational | 1 – 3 prior regimens; 38 % had 1, 41 % had 2, 21 % had 3 | PLD 50 mg/m² IV q4w + Trebananib 15 mg/kg IV weekly |
| Joly (2022) | Pazopanib | Investigational | ≥ 1 prior platinum regimen; 76 – 78% had 1 line, 22 – 24% had 2 lines | Paclitaxel 65 mg/m² IV D1, 8, 15 q28d + Pazopanib 600 mg/day (up to 800 mg if tolerated) vs Paclitaxel 80 mg/m² IV D1, 8, 15 q28d |
