# Supplementary material for: Molecular targeted therapy in combination with chemotherapy for the treatment of platinum-resistant/refractory ovarian cancer (PROC): a systematic review and network meta-analysis
Source: Ann Med. 2026 Feb 23;58(1):2624215. doi: 10.1080/07853890.2026.2624215 (PMC12931348; doi:10.1080/07853890.2026.2624215)
Supplement: Supplementary Table S1.docx [file IANN_A_2624215_SM0125.docx]

**Supplementary Table S1.** Literature search strategy.

**1.PubMed**

| Search number | Query |
| --- | --- |
| #1 | "Ovarian Neoplasms"[Title/Abstract] OR "ovary cancer"[Title/Abstract] OR "Cancer of Ovary"[Title/Abstract] OR "Cancer of the Ovary"[Title/Abstract] OR "carcinogenesis of the ovary"[Title/Abstract] OR "malignancies of the ovary"[Title/Abstract] OR "malignancy of the ovary"[Title/Abstract] OR "malignant neoplasm of the ovary"[Title/Abstract] OR "malignant neoplasms of the ovary"[Title/Abstract] OR "malignant ovarian neoplasm"[Title/Abstract] OR "malignant ovarian tumor"[Title/Abstract] OR "malignant ovarian tumour"[Title/Abstract] OR "malignant ovary tumor"[Title/Abstract] OR "malignant ovary tumour"[Title/Abstract] OR "malignant tumor of the ovary"[Title/Abstract] OR "malignant tumors of the ovary"[Title/Abstract] OR "ovarial cancer"[Title/Abstract] OR "ovarian Ca"[Title/Abstract] OR "ovarian cancer"[Title/Abstract] OR "ovarian cancerogenesis"[Title/Abstract] OR "Ovarian Cancers"[Title/Abstract] OR "ovarian carcinogenesis"[Title/Abstract] OR "ovarian malignancies"[Title/Abstract] OR "ovarian malignancy"[Title/Abstract] OR "Ovarian Neoplasm"[Title/Abstract] OR "Ovarian Neoplasms"[Title/Abstract] OR "ovarium cancer"[Title/Abstract] OR "ovary cancer"[Title/Abstract] OR "Ovary Cancers"[Title/Abstract] OR "ovary carcinogenesis"[Title/Abstract] OR "Ovary Neoplasm"[Title/Abstract] OR "Ovary Neoplasms"[Title/Abstract] |
| #2 | ovarian neoplasms[MeSH Terms] |
| #3 | "platinum resistant"[Title/Abstract] OR "platinum-resistant"[Title/Abstract] OR "platinum refractory"[Title/Abstract] OR "platinum-refractory"[Title/Abstract] |
| #4 | random*[Title/Abstract] OR control*[Title/Abstract] |
| #5 | (#1 OR #2) AND #3 AND #4 384 |

**2.Cochrane**

| Search number | Query |
| --- | --- |
| #1 | (' Ovarian Neoplasms' OR ' ovary cancer' OR ' Cancer of Ovary' OR ' Cancer of the Ovary' OR ' carcinogenesis of the ovary' OR ' malignancies of the ovary' OR ' malignancy of the ovary' OR ' malignant neoplasm of the ovary' OR ' malignant neoplasms of the ovary' OR ' malignant ovarian neoplasm' OR ' malignant ovarian tumor' OR ' malignant ovarian tumour' OR ' malignant ovary tumor' OR ' malignant ovary tumour' OR ' malignant tumor of the ovary' OR ' malignant tumors of the ovary' OR ' ovarial cancer' OR ' ovarian Ca' OR ' ovarian cancer' OR ' ovarian cancerogenesis' OR ' Ovarian Cancers' OR ' ovarian carcinogenesis' OR ' ovarian malignancies' OR ' ovarian malignancy' OR ' Ovarian Neoplasm' OR ' Ovarian Neoplasms' OR ' ovarium cancer' OR ' ovary cancer' OR ' Ovary Cancers' OR ' ovary carcinogenesis' OR ' Ovary Neoplasm' OR ' Ovary Neoplasms'):ti,ab,kw 9427 |
| #2 | MeSH descriptor: [Ovarian Neoplasms] explode all trees 2960 |
| #3 | (' platinum resistant' OR ' platinum-resistant' OR ' platinum refractory' OR ' platinum-refractory):ti,ab,kw 1389 |
| #4 | (random* OR control*):ti,ab,kw 1498338 |
| #5 | (#1 OR #2) AND #3 AND #4 644 |

**3.Embase**

| Search number | Query |
| --- | --- |
| #1 | 'cancer of ovary':ti,ab,kw OR 'cancer of the ovary':ti,ab,kw OR 'carcinogenesis of the ovary':ti,ab,kw OR 'malignancies of the ovary':ti,ab,kw OR 'malignancy of the ovary':ti,ab,kw OR 'malignant neoplasm of the ovary':ti,ab,kw OR 'malignant neoplasms of the ovary':ti,ab,kw OR 'malignant ovarian neoplasm':ti,ab,kw OR 'malignant ovarian tumor':ti,ab,kw OR 'malignant ovarian tumour':ti,ab,kw OR 'malignant ovary tumor':ti,ab,kw OR 'malignant ovary tumour':ti,ab,kw OR 'malignant tumor of the ovary':ti,ab,kw OR 'malignant tumors of the ovary':ti,ab,kw OR 'ovarial cancer':ti,ab,kw OR 'ovarian ca':ti,ab,kw OR 'ovarian cancer':ti,ab,kw OR 'ovarian cancerogenesis':ti,ab,kw OR 'ovarian cancers':ti,ab,kw OR 'ovarian carcinogenesis':ti,ab,kw OR 'ovarian malignancies':ti,ab,kw OR 'ovarian malignancy':ti,ab,kw OR 'ovarian neoplasm':ti,ab,kw OR 'ovarian neoplasms':ti,ab,kw OR 'ovarium cancer':ti,ab,kw OR 'ovary cancer':ti,ab,kw OR 'ovary cancers':ti,ab,kw OR 'ovary carcinogenesis':ti,ab,kw OR 'ovary neoplasm':ti,ab,kw OR 'ovary neoplasms':ti,ab,kw |
| #2 | 'platinum resistant':ti,ab,kw OR 'platinum-resistant':ti,ab,kw OR 'platinum refractory':ti,ab,kw OR 'platinum-refractory':ti,ab,kw |
| #3 | 'ovary cancer'/exp |
| #4 | (#1 OR #3) AND #2 |
| #5 | #4 AND 'Article'/it 1476 |

**4.Web of science**

| Search number | Query |
| --- | --- |
| #1 | Ovarian Neoplasms (Topic) or Ovarian Neoplasm (Topic) or Ovary Neoplasms (Topic) or Neoplasm, Ovary (Topic) or Neoplasms, Ovary (Topic) or Ovary Neoplasm (Topic) |
| #2 | Recurrence (Topic) or Recurrences (Topic) or Recrudescence (Topic) or Recrudescences (Topic) or Relapse (Topic) or Relapses (Topic) |
| #3 | Neoplasms, Ovarian (Topic) or Ovary Cancer (Topic) or Cancer, Ovary (Topic) or Cancers, Ovary (Topic) or Ovary Cancers (Topic) or Cancer, Ovarian (Topic) or Cancers, Ovarian (Topic) |
| #4 | Ovarian Cancer (Topic) or Ovarian Cancers (Topic) or Cancer of Ovary (Topic) or Cancer of the Ovary (Topic) or ovary tumor (Topic) |
| #5 | #1 AND #2 AND #3 AND #4 729 |
