# Supplementary material for: Molecular targeted therapy in combination with chemotherapy for the treatment of platinum-resistant/refractory ovarian cancer (PROC): a systematic review and network meta-analysis
Source: Ann Med. 2026 Feb 23;58(1):2624215. doi: 10.1080/07853890.2026.2624215 (PMC12931348; doi:10.1080/07853890.2026.2624215)
Supplement: Supplementary Table S4.docx [file IANN_A_2624215_SM0124.docx]

**Supplementary Table S4.** Summary of chemotherapy backbones and targeted agents across included studies

| Study ID | Chemotherapy backbone | Targeted agent |
| --- | --- | --- |
| Banerjee (2022) | Paclitaxel (weekly) | Vistusertib |
| Sharma (2021) | Etoposide + Cyclophosphamide | Pazopanib |
| Liu (2016) | Paclitaxel (weekly) | Seribantumab |
| Lheureux (2021) | Gemcitabine | Adavosertib |
| Pignata 2016 | Paclitaxel (weekly) | Pazopanib |
| McNeish 2014 | Paclitaxel (weekly) | Saracatinib |
| Duska 2019 | Gemcitabine (weekly) | Pazopanib |
| Naumann (2013) | Pegylated liposomal doxorubicin (PLD) | Vintafolide (EC145) |
| Pujade-Lauraine (2021) | Pegylated liposomal doxorubicin (PLD) | Avelumab |
| Konstantinopoulos (2020) | Gemcitabine | Berzosertib |
| Pujade-Lauraine (2014) | Paclitaxel / PLD / Topotecan | Bevacizumab |
| Lee (2022) | PLD / Topotecan / Paclitaxel (weekly) | Olaparib / Cediranib / Durvalumab / Tremelimumab |
| Makhija (2010) | Gemcitabine | Pertuzumab |
| Kurzeder (2016) | Topotecan / Paclitaxel (weekly) / Gemcitabine | Pertuzumab |
| Shoji (2021) | Pegylated liposomal doxorubicin (PLD) / Topotecan / Paclitaxel / Gemcitabine | Bevacizumab |
| Liu (2019) | Albumin-bound paclitaxel | Bevacizumab |
| Oza (2018) | Paclitaxel (weekly) | Linsitinib |
| Roque (2022) | Ixabepilone | Bevacizumab |
| McGuire (2018) | Liposomal doxorubicin (PLD) | Olaratumab |
| Chekerov (2018) | Topotecan | Sorafenib |
| Marth (2017) | Pegylated liposomal doxorubicin (PLD) | Trebananib |
| Joly (2022) | Paclitaxel (weekly) | Pazopanib |
