# Supplementary material for: Molecular targeted therapy in combination with chemotherapy for the treatment of platinum-resistant/refractory ovarian cancer (PROC): a systematic review and network meta-analysis
Source: Ann Med. 2026 Feb 23;58(1):2624215. doi: 10.1080/07853890.2026.2624215 (PMC12931348; doi:10.1080/07853890.2026.2624215)
Supplement: Supplementary Table S7.docx [file IANN_A_2624215_SM0123.docx]

**Supplementary Table S7.** Details of Grade 3-4 AEs in included trials.

| Study | Grade 3-4 AEs n (%) | | | | | | | | | | | | | |
| --- | --- | --- | --- | --- | --- | --- | --- | --- | --- | --- | --- | --- | --- | --- |
| Banerjee (2022) |  | Weekly paclitaxel + Vistusertib (wP+V):  Grade 3 | | | Weekly paclitaxel + Vistusertib (wP+V):  Grade 4 | | | | Weekly paclitaxel + Placebo (wP+P):  Grade 3 | | | Weekly paclitaxel + Placebo (wP+P):  Grade 4 | | |
|  | Alopecia | 1 (1.5%) | | | 0 | | | | 1 (1.5%) | | | 0 | | |
|  | Diarrhoea | 2 (2.9%) | | | 0 | | | | 1 (1.5%) | | | 0 | | |
|  | Dyspnea | 3 (4.4%) | | | 0 | | | | 0 | | | 0 | | |
|  | Fatigue | 6 (8.8%) | | | 0 | | | | 3 (4.4%) | | | 0 | | |
|  | Nausea | 1 (1.5%) | | | 0 | | | | 0 | | | 0 | | |
|  | Rash maculo-papular | 1 (1.5%) | | | 0 | | | | 0 | | | 0 | | |
|  | Vomiting | 1 (1.5%) | | | 0 | | | | 1 (1.5%) | | | 0 | | |
|  | Haemoglobin | 1 (1.5%) | | | 0 | | | | 0 | | | 0 | | |
|  | Lymphocytes | 8 (11.8%) | | | 1 (1.5%) | | | | 1 (1.5%) | | | 0 | | |
|  | Neutrophils | 4 (5.9%) | | | 1 (1.5%) | | | | 1 (1.5%) | | | 0 | | |
|  | WBC | 4 (5.9%) | | | 1 (1.5%) | | | | 0 | | | 0 | | |
|  | Albumin | 4 (5.9%) | | | 0 | | | | 1 (1.5%) | | | 0 | | |
|  | Bilirubin | 4 (5.9%) | | | 0 | | | | 1 (1.5%) | | | 0 | | |
|  | Magnesium (low) | 2 (2.9%) | | | 0 | | | | 0 | | | 0 | | |
|  | Phosphate | 2 (2.9%) | | | 0 | | | | 1 (1.5%) | | | 0 | | |
| Sharma (2021) | Grade 3–4 | Oral etoposide + cyclophosphamide | | | | | | | Pazopanib +oral etoposide + cyclophosphamide | | | | | |
|  | Mucositis | 4 (10.5%) | | | | | | | 11 (29.7%) | | | | | |
|  | Fatigue | 2 (5.2%) | | | | | | | 5 (13.5%) | | | | | |
|  | Hypertension | 0 | | | | | | | 2 (5.4%) | | | | | |
|  | Vomiting | 3 (7.8%) | | | | | | | 2 (5.4%) | | | | | |
|  | Nausea | 4 (10.5%) | | | | | | | 2 (5.4%) | | | | | |
|  | **Hematological** |  | | | | | | |  | | | | | |
|  | Anaemia | 4 (10.5%) | | | | | | | 6 (16.2%) | | | | | |
|  | Thrombocytopenia | 2 (5.2%) | | | | | | | 2 (5.4%) | | | | | |
|  | Neutropenia | 3 (7.8%) | | | | | | | 7 (18.9%) | | | | | |
|  | Liver dysfunction | 0 | | | | | | | 2 (5.2%) | | | | | |
|  | Hypothyroidism | 0 | | | | | | | 1 (2.7%) | | | | | |
|  | Hand–foot skin reaction | 0 | | | | | | | 3 (7.9%) | | | | | |
| Liu (2016 | Grade ≥3 | Seribantumab + Paclitaxel | | | | | | | Paclitaxel | | | | | |
|  | At least one TEAE | 85 (60.7%) | | | | | | | 43 (53.8%) | | | | | |
|  | Diarrhea | 10 (7.1%) | | | | | | | 4 (5.0%) | | | | | |
|  | Fatigue | 11 (7.9%) | | | | | | | 4 (5.0%) | | | | | |
|  | Nausea | 5 (3.6%) | | | | | | | 3 (3.8%) | | | | | |
|  | Abdominal pain | 2 (1.4%) | | | | | | | 3 (3.8%) | | | | | |
|  | Vomiting | 2 (1.4%) | | | | | | | 3 (3.8%) | | | | | |
|  | Anemia | 18 (12.9%) | | | | | | | 7 (8.8%) | | | | | |
|  | Decreased appetite | 1 (0.7%) | | | | | | | 2 (2.5%) | | | | | |
|  | Rash | 1 (0.7%) | | | | | | | 1 (1.3%) | | | | | |
|  | Asthenia | 3 (2.1%) | | | | | | | 2 (2.5%) | | | | | |
|  | Hypokalemia | 11 (7.9%) | | | | | | | 0 | | | | | |
|  | Edema | 2 (1.4%) | | | | | | | 0 | | | | | |
|  | Stomatitis | 2 (1.4%) | | | | | | | 0 | | | | | |
|  | Mucosal inflammation | 2 (1.4%) | | | | | | | 0 | | | | | |
|  | Hypomagnesemia | 3 (2.1%) | | | | | | | 0 | | | | | |
|  | Constipation | 0 | | | | | | | 1 (1.3%) | | | | | |
|  | Peripheral neuropathy | 2 (1.4%) | | | | | | | 1 (1.3%) | | | | | |
|  | Pyrexia | 6 (4.3%) | | | | | | | 2 (2.5%) | | | | | |
|  | Cough | 0 | | | | | | | 0 | | | | | |
|  | Dyspnea | 6 (4.3%) | | | | | | | 2 (2.5%) | | | | | |
|  | Neutropenia | 9 (6.4%) | | | | | | | 8 (10.0%) | | | | | |
|  | Urinary tract infection | 3 (2.1%) | | | | | | | 1 (1.3%) | | | | | |
|  | Sensory neuropathy | 1 (0.7%) | | | | | | | 1 (1.3%) | | | | | |
|  | Headache | 1 (0.7%) | | | | | | | 0 | | | | | |
|  | Anxiety | 1 (0.7%) | | | | | | | 0 | | | | | |
|  | Hyperglycemia | 3 (2.1%) | | | | | | | 3 (3.8%) | | | | | |
|  | Dehydration | 3 (2.1%) | | | | | | | 0 | | | | | |
|  | Back pain | 3 (2.1%) | | | | | | | 0 | | | | | |
| Lheureux (2021) | Grade ≥3 | Adavosertib + Gemcitabine | | | | | | | Placebo + Gemcitabine | | | | | |
|  | Fatigue | 10 (16%) | | | | | | | 3 (9%) | | | | | |
|  | Anaemia | 19 (31%) | | | | | | | 7 (21%) | | | | | |
|  | Decreased white blood cell count | 33 (54%) | | | | | | | 6 (18%) | | | | | |
|  | Decreased platelet count (thrombocytopenia) | 19 (31%) | | | | | | | 2 (6%) | | | | | |
|  | Decreased neutrophil count (neutropenia) | 38 (62%) | | | | | | | 10 (30%) | | | | | |
|  | Nausea | 2 (3%) | | | | | | | 2 (6%) | | | | | |
|  | Decreased lymphocyte count | 21 (34%) | | | | | | | 6 (18%) | | | | | |
|  | Abdominal pain | 5 (8%) | | | | | | | 4 (12%) | | | | | |
|  | Hypoalbuminaemia | 1 (2%) | | | | | | | 0 | | | | | |
|  | Increased alanine aminotransferase | 2 (3%) | | | | | | | 2 (6%) | | | | | |
|  | Increased aspartate aminotransferase | 4 (7%) | | | | | | | 3 (9%) | | | | | |
|  | Diarrhoea | 4 (7%) | | | | | | | 1 (3%) | | | | | |
|  | Hypertension | 9 (15%) | | | | | | | 1 (3%) | | | | | |
|  | Vomiting | 1 (2%) | | | | | | | 3 (9%) | | | | | |
|  | Dyspnoea | 4 (7%) | | | | | | | 3 (9%) | | | | | |
|  | Hyponatraemia | 2 (3%) | | | | | | | 2 (6%) | | | | | |
|  | Back pain | 2 (3%) | | | | | | | 0 | | | | | |
|  | Maculopapular rash | 4 (7%) | | | | | | | 0 | | | | | |
|  | Limb oedema | 1 (2%) | | | | | | | 0 | | | | | |
|  | Pruritus | 1 (2%) | | | | | | | 0 | | | | | |
|  | Headache | 1 (2%) | | | | | | | 0 | | | | | |
|  | Hypokalaemia | 6 (10%) | | | | | | | 3 (9%) | | | | | |
|  | Weight loss | 0 | | | | | | | 1 (3%) | | | | | |
|  | Increased alkaline phosphatase | 1 (2%) | | | | | | | 0 | | | | | |
|  | Hypophosphataemia | 4 (7%) | | | | | | | 0 | | | | | |
|  | Glucose intolerance | 1 (2%) | | | | | | | 0 | | | | | |
|  | Thromboembolic event | 5 (8%) | | | | | | | 4 (12%) | | | | | |
|  | Febrile neutropenia | 7 (11%) | | | | | | | 0 | | | | | |
|  | Dehydration | 1 (2%) | | | | | | | 1 (3%) | | | | | |
| Pignata 2016 | Adverse Event | Paclitaxel G3 | | | Paclitaxel G4 | | | | Paclitaxel + Pazopanib G3 | | | Paclitaxel + Pazopanib G4 | | |
|  | Anemia | 5 (14%) | | | 0 | | | | 2 (5%) | | | 0 | | |
|  | Leucopenia | 1 (3%) | | | 0 | | | | 4 (11%) | | | 0 | | |
|  | Neutropenia | 1 (3%) | | | 0 | | | | 8 (22%) | | | 3 (8%) | | |
|  | Febrile Neutropenia | 0 | | | 0 | | | | 2 (5%) | | | 0 | | |
|  | Hypertension | 0 | | | 0 | | | | 3(8%) | | | 0 | | |
|  | Heart general | 0 | | | 0 | | | | 1 (3%) | | | 0 | | |
|  | Thromboembolic event | 1 (3%) | | | 0 | | | | 1 (3%) | | | 0 | | |
|  | Fatigue | 2 (6%) | | | 0 | | | | 4 (11%) | | | 0 | | |
|  | Constipation | 1 (3%) | | | 0 | | | | 1 (3%) | | | 0 | | |
|  | Diarrhoea | 0 | | | 0 | | | | 2 (5%) | | | 0 | | |
|  | Vomiting | 0 | | | 0 | | | |  | | | 0 | | |
|  | ALP | 0 | | | 0 | | | | 1 (3%) | | | 0 | | |
|  | AST/ALT | 0 | | | 0 | | | | 2 (5%) | | | 0 | | |
|  | Gamma-GT | 0 | | | 0 | | | | 1 (3%) | | | 0 | | |
|  | Lipase | 0 | | | 0 | | | | 1 (3%) | | | 0 | | |
|  | Amylase | 0 | | | 0 | | | | 1 (3%) | | | 0 | | |
|  | Neurology other | 0 | | | 0 | | | | 3 (8%) | | | 0 | | |
| McNeish 2014 | Grade ≥3 | Saracatinib | | | | | | | Placebo | | | | | |
|  | Anaemia | 1 (1.4%) | | | | | | | 0 | | | | | |
|  | Febrile neutropenia | 3 (4.3%) | | | | | | | 0 | | | | | |
|  | Neutrophil count decreased | 2 (2.9%) | | | | | | | 0 | | | | | |
|  | Vomiting | 4 (5.8%) | | | | | | | 3 (8.6%) | | | | | |
|  | Abdominal pain | 4 (5.8%) | | | | | | | 0 | | | | | |
|  | Diarrhoea | 3 (4.3%) | | | | | | | 2 (5.7%) | | | | | |
|  | Fever | 3 (4.3%) | | | | | | | 1 (2.9%) | | | | | |
|  | Small intestinal obstruction | 3 (4.3%) | | | | | | | 1 (2.9%) | | | | | |
|  | Dyspnoea | 2 (2.9%) | | | | | | | 1 (2.9%) | | | | | |
|  | Back pain | 0 | | | | | | | 1 (2.9%) | | | | | |
|  | Catheter-related infection | 0 | | | | | | | 1 (2.9%) | | | | | |
|  | Fatigue | 2 (2.9%) | | | | | | | 1 (2.9%) | | | | | |
|  | Abdominal distension | 0 | | | | | | | 1 (2.9%) | | | | | |
|  | Dehydration | 2 (2.9%) | | | | | | | 0 | | | | | |
|  | PPES | 0 | | | | | | | 1 (2.9%) | | | | | |
|  | Rash maculo-papular | 2 (2.9%) | | | | | | | 1 (2.9%) | | | | | |
|  | Skin infection | 0 | | | | | | | 1 (2.9%) | | | | | |
|  | Nausea | 1 (1.4%) | | | | | | | 1 (2.9%) | | | | | |
|  | Urinary tract infection | 1 (1.4%) | | | | | | | 1 (2.9%) | | | | | |
|  | Anorexia | 1 (1.4%) | | | | | | | 0 | | | | | |
|  | Ventricular arrhythmia | 0 | | | | | | | 1 (2.9%) | | | | | |
|  | Creatinine increased | 1 (1.4%) | | | | | | | 0 | | | | | |
|  | Endocarditis infective | 1 (1.4%) | | | | | | | 0 | | | | | |
|  | Fungal chest infection | 1 (1.4%) | | | | | | | 0 | | | | | |
|  | Lung infection | 1 (1.4%) | | | | | | | 0 | | | | | |
|  | Myocardial infarction | 1 (1.4%) | | | | | | | 0 | | | | | |
|  | Pneumonitis | 1 (1.4%) | | | | | | | 0 | | | | | |
| Duska 2019 | grades 3-5 | Gemcitabine | | | | | | | Gemcitabine + Pazopanib | | | | | |
|  | Anemia | 2 (3%) | | | | | | | 8 (11%) | | | | | |
|  | Neutropenia | 15 (20%) | | | | | | | 30 (40%) | | | | | |
|  | Thrombocytopenia | 2 (3%) | | | | | | | 11 (15%) | | | | | |
|  | Fatigue | 1 (1%) | | | | | | | 9 (12%) | | | | | |
|  | Elevated AST | 1 (1%) | | | | | | | 9 (12%) | | | | | |
|  | Hypertension | 1 (1%) | | | | | | | 12 (16%) | | | | | |
| Naumann (2013) | Grade 3–4 | Vintafolide + PLD | | | | | | | PLD | | | | | |
|  | Anemia | 9 | | | | | | | 8 | | | | | |
|  | Leukopenia | 9 | | | | | | | 0 | | | | | |
|  | Neutropenia | 23 | | | | | | | 10 | | | | | |
|  | Febrile neutropenia | 1 | | | | | | | 2 | | | | | |
|  | Thrombocytopenia | 4 | | | | | | | 4 | | | | | |
|  | Fatigue | 9 | | | | | | | 6 | | | | | |
|  | Constipation | 2 | | | | | | | 0 | | | | | |
|  | Peripheral sensory neuropathy | 4 | | | | | | | 0 | | | | | |
|  | Abdominal pain | 8 | | | | | | | 2 | | | | | |
|  | Nausea | 1 | | | | | | | 8 | | | | | |
|  | Stomatitis | 8 | | | | | | | 4 | | | | | |
|  | Palmar-plantar erythrodysesthesia syndrome | 11 | | | | | | | 2 | | | | | |
| Pujade-Lauraine (2021) |  | Avelumab + PLD G3 | Avelumab + PLD G4 | | | | PLD  G3 | | PLD  G4 | | Avelumab G3 | | | Avelumab G4 |
|  | Any treatment-related AE | 70 (38%) | 8 (4%) | | | | 47 (27%) | | 8 (5%) | | 25 (13%) | | | 4 (2%) |
|  | Nausea | 3 (2%) | 0 | | | | 1 (1%) | | 0 | | 0 | | | 0 |
|  | Fatigue | 10 (5%) | 0 | | | | 3 (2%) | | 0 | | 0 | | | 0 |
|  | Palmar-plantar erythrodysesthesia syndrome | 18 (10%) | 0 | | | | 9 (5%) | | 0 | | 0 | | | 0 |
|  | Stomatitis | 10 (5%) | 0 | | | | 4 (2%) | | 1 (1%) | | 0 | | | 0 |
|  | Rash | 11 (6%) | 0 | | | | 3 (2%) | | 0 | | 0 | | | 0 |
|  | Anaemia | 6 (3%) | 0 | | | | 9 (5%) | | 0 | | 3 (2%) | | | 0 |
|  | Decreased appetite | 1 (1%) | 0 | | | | 0 | | 0 | | 0 | | | 0 |
|  | Pyrexia | 0 | 0 | | | | 0 | | 0 | | 0 | | | 0 |
|  | Mucosal inflammation | 3 (2%) | 0 | | | | 3 (2%) | | 0 | | 1 (1%) | | | 0 |
|  | Vomiting | 1 (1%) | 0 | | | | 3 (2%) | | 0 | | 1 (1%) | | | 0 |
|  | Pruritus | 0 | 0 | | | | 0 | | 0 | | 0 | | | 0 |
|  | Diarrhoea | 1 (1%) | 0 | | | | 0 | | 0 | | 5 (3%) | | | 2 (1%) |
|  | Infusion-related reaction | 1 (1%) | 0 | | | | 0 | | 1 (1%) | | 0 | | | 0 |
|  | Asthenia | 4 (2%) | 0 | | | | 1 (1%) | | 0 | | 0 | | | 0 |
|  | Neutropenia | 7 (4%) | 2 (1%) | | | | 7 (4%) | | 1 (1%) | | 0 | | | 0 |
|  | Constipation | 0 | 0 | | | | 0 | | 0 | | 0 | | | 0 |
|  | White blood cell count decreased | 5 (3%) | 1 (1%) | | | | 4 (2%) | | 1 (1%) | | 0 | | | 0 |
|  | Neutrophil count decreased | 7 (4%) | 1 (1%) | | | | 3 (2%) | | 1 (1%) | | 0 | | | 0 |
|  | Rash maculopapular | 5 (3%) | 0 | | | | 1 (1%) | | 0 | | 0 | | | 0 |
|  | Lymphocyte count decreased | 5 (3%) | 0 | | | | 1 (1%) | | 0 | | 0 | | | 0 |
|  | Hyponatraemia | 1 (1%) | 0 | | | | 0 | | 0 | | 3 (2%) | | | 0 |
|  | Leukopenia | 1 (1%) | 0 | | | | 3 (2%) | | 0 | | 0 | | | 0 |
|  | Febrile neutropenia | 0 | 1 (1%) | | | | 1 (1%) | | 0 | | 0 | | | 0 |
| Konstantinopoulos (2020) |  | Gemcitabine G3 | | | Gemcitabine G4 | | | | Gemcitabine + Berzosertib G3 | | | Gemcitabine + Berzosertib G4 | | |
|  | Anaemia | 4 (11%) | | | 0 | | | | 5 (15%) | | | 0 | | |
|  | Febrile neutropenia | 1 (3%) | | | 0 | | | | 1 (3%) | | | 0 | | |
|  | Haemolytic uraemic syndrome | 1 (3%) | | | 0 | | | | 0 | | | 0 | | |
|  | Thrombocytosis | 0 | | | 0 | | | | 1 (3%) | | | 0 | | |
|  | Lymphocyte count decreased | 1 (3%) | | | 0 | | | | 1 (3%) | | | 0 | | |
|  | Neutrophil count decreased | 10 (28%) | | | 4 (11%) | | | | 12 (35%) | | | 4 (12%) | | |
|  | Platelet count decreased | 0 | | | 2 (6%) | | | | 2 (6%) | | | 6 (18%) | | |
|  | White blood cell decreased | 4 (11%) | | | 0 | | | | 3 (9%) | | | 0 | | |
|  | Myocardial infarction | 1 (3%) | | | 0 | | | | 0 | | | 0 | | |
|  | Abdominal pain | 0 | | | 0 | | | | 1 (3%) | | | 0 | | |
|  | Diarrhoea | 0 | | | 0 | | | | 1 (3%) | | | 0 | | |
|  | Gastric haemorrhage | 1 (3%) | | | 0 | | | | 0 | | | 0 | | |
|  | Nausea | 1 (3%) | | | 0 | | | | 1 (3%) | | | 0 | | |
|  | Vomiting | 0 | | | 0 | | | | 1 (3%) | | | 0 | | |
|  | Oedema limbs | 1 (3%) | | | 0 | | | | 0 | | | 0 | | |
|  | Fatigue | 3 (8%) | | | 0 | | | | 3 (9%) | | | 0 | | |
|  | Fall | 1 (3%) | | | 0 | | | | 0 | | | 0 | | |
|  | Cardiac troponin T increased | 1 (3%) | | | 0 | | | | 0 | | | 0 | | |
|  | Generalised muscle weakness | 0 | | | 0 | | | | 1 (3%) | | | 0 | | |
|  | Headache | 0 | | | 0 | | | | 1 (3%) | | | 0 | | |
|  | Dyspnoea | 1 (3%) | | | 0 | | | | 0 | | | 0 | | |
|  | Capillary leak syndrome | 1 (3%) | | | 0 | | | | 0 | | | 0 | | |
| Pujade-Lauraine (2014) | Grade ≥3 | Chemotherapy Alone | | | | | | | Bevacizumab + Chemotherapy | | | | | |
|  | Hypertension | 2 (1%) | | | | | | | 13 (7%) | | | | | |
|  | Proteinuria | 0 | | | | | | | 3 (2%) | | | | | |
|  | GI perforation | 0 | | | | | | | 3 (2%) | | | | | |
|  | Fistula/abscess | 0 | | | | | | | 2 (1%) | | | | | |
|  | Bleeding | 2 (1%) | | | | | | | 2 (1%) | | | | | |
|  | Thromboembolic event | 8 (4%) | | | | | | | 9 (5%) | | | | | |
|  | └ Arterial | 0 | | | | | | | 4 (2%) | | | | | |
|  | └ Venous | 8 (4%) | | | | | | | 5 (3%) | | | | | |
|  | Wound-healing complication | 0 | | | | | | | 0 | | | | | |
|  | Reversible posterior leukoencephalopathy syndrome | 0 | | | | | | | 1 (1%) | | | | | |
|  | Congestive heart failure | 1 (1%) | | | | | | | 1 (1%) | | | | | |
|  | Cardiac disorders (excluding congestive heart failure) | 0 | | | | | | | 0 | | | | | |
| Lee (2022) | Grade 3–4 | olaparib + cediranib | | olaparib + durvalumab | | | | durvalumab + CT) | | durvalumab + tremelimumab 75 mg+ CT) | | | durvalumab + tremelimumab 300 mg + CT | |
|  | Neutrophil count decreased | 18.8% | | 21.4% | | | | 20.0% | | 50.0% | | | 17.65% | |
|  | Anaemia | 18.8% | | 21.4% | | | | 16.7% | | 5.9% | | | 14.3% | |
|  | Platelet count decreased | 6.25% | | 7.14% | | | | 0 | | 0 | | | 0 | |
|  | Alanine aminotransferase increased | 6.25% | | 0 | | | | 0 | | 0 | | | 0 | |
|  | Aspartate aminotransferase increased | 0 | | 0 | | | | 0 | | 11.1% | | | 0 | |
|  | Lipase increased | 0 | | 0 | | | | 0 | | 11.1% | | | 0 | |
|  | Febrile neutropenia | 0 | | 0 | | | | 0 | | 16.7% | | | 0 | |
| Makhija (2010) | Grade 3 or 4 | Gemcitabine + Pertuzumab | | | | | | | Gemcitabine | | | | | |
|  | Neutropenia | 23 (35%) | | | | | | | 14 (22%) | | | | | |
|  | Thrombocytopenia | 9 (14%) | | | | | | | 5 (8%) | | | | | |
|  | Anemia | 3 (5%) | | | | | | | 3 (5%) | | | | | |
|  | Fatigue | 14 (22%) | | | | | | | 11 (17%) | | | | | |
|  | Nausea | 5 (8%) | | | | | | | 4 (6%) | | | | | |
|  | Diarrhea | 7 (11%) | | | | | | | 1 (2%) | | | | | |
|  | Back pain | 6 (9%) | | | | | | | 1 (2%) | | | | | |
|  | Headache | 1 (2%) | | | | | | | 1 (2%) | | | | | |
|  | Dyspepsia | 1 (2%) | | | | | | | 0 | | | | | |
|  | Congestive heart failure | 1 (2%) | | | | | | | 0 | | | | | |
| Kurzeder (2016) | Adverse Event (≥Grade 3) | Pertuzumab + Chemo | | | | | | | Placebo + Chemo | | | | | |
|  | **Diarrhea** | 11 (14.3%) | | | | | | | 1 (1.3%) | | | | | |
|  | **Fatigue/asthenia** | 6 (7.8%) | | | | | | | 9 (11.8%) | | | | | |
|  | **Nausea** | 4 (5.2%) | | | | | | | 1 (1.3%) | | | | | |
|  | **Neutropenia** | 24 (31.2%) | | | | | | | 16 (21.1%) | | | | | |
|  | **Anemia** | 6 (7.8%) | | | | | | | 5 (6.6%) | | | | | |
|  | **Vomiting** | 4 (5.2%) | | | | | | | 2 (2.6%) | | | | | |
|  | **Abdominal pain** | 2 (2.6%) | | | | | | | 2 (2.6%) | | | | | |
|  | **Alopecia** | 1 (1.3%) | | | | | | | 1 (1.3%) | | | | | |
|  | **Constipation** | 0 | | | | | | | 1 (1.3%) | | | | | |
|  | **Leukopenia** | 5 (6.5%) | | | | | | | 7 (9.2%) | | | | | |
|  | **Hypokalemia** | 5 (6.5%) | | | | | | | 4 (5.3%) | | | | | |
|  | **Thrombocytopenia** | 4 (5.2%) | | | | | | | 3 (3.9%) | | | | | |
|  | **Hypertension** | 4 (5.2%) | | | | | | | 3 (3.9%) | | | | | |
|  | **GGT increased** | 2 (2.6%) | | | | | | | 4 (5.3%) | | | | | |
|  | **Intestinal obstruction** | 1 (1.3%) | | | | | | | 5 (6.6%) | | | | | |
|  | **Febrile neutropenia** | 2 (2.6%) | | | | | | | 4 (5.3%) | | | | | |
| Shoji (2021) | Adverse Event (≥Grade 3) | Chemo | | | | | | | Chemo + BEV | | | | | |
|  | **Any ≥3 AE** | 23 (46.0%) | | | | | | | 30 (58.8%) | | | | | |
|  | **Any ≥3 treatment-related AE** | 21 (42.0%) | | | | | | | 28 (54.9%) | | | | | |
|  | Neutrophil count decreased | 16 (32.0%) | | | | | | | 19 (37.3%) | | | | | |
|  | Platelet count decreased | 7 (14.0%) | | | | | | | 5 (9.8%) | | | | | |
|  | Anemia | 4 (8.0%) | | | | | | | 5 (9.8%) | | | | | |
|  | Febrile neutropenia | 3 (6.0%) | | | | | | | 1 (2.0%) | | | | | |
|  | Anorexia | 1 (2.0%) | | | | | | | 3 (5.9%) | | | | | |
|  | Palmar–plantar erythrodysesthesia | 2 (4.0%) | | | | | | | 0 | | | | | |
|  | Infections/infestations (other) | 0 | | | | | | | 3 (5.9%) | | | | | |
|  | Proteinuria | 0 | | | | | | | 3 (5.9%) | | | | | |
|  | Hypertension | 0 | | | | | | | 2 (3.9%) | | | | | |
|  | Oral mucositis | 0 | | | | | | | 2 (3.9%) | | | | | |
|  | Hypoalbuminemia | 0 | | | | | | | 2 (3.9%) | | | | | |
| Liu (2019) | Adverse Event (Grade III–IV) | BEV+ABP | | | | | | | ABP | | | | | |
|  | Fever | 0 (0%) | | | | | | | 0 (0%) | | | | | |
|  | Fatigue | 2 (4.7%) | | | | | | | 1 (2.3%) | | | | | |
|  | Erythema | 0 (0%) | | | | | | | 0 (0%) | | | | | |
|  | Alopecia | 0 (0%) | | | | | | | 0 (0%) | | | | | |
|  | Neutropenia | 4 (9.3%) | | | | | | | 2 (4.7%) | | | | | |
|  | Thrombocytopenia | 5 (11.6%) | | | | | | | 2 (4.7%) | | | | | |
|  | Anemia | 0 (0%) | | | | | | | 0 (0%) | | | | | |
|  | Nausea/Vomiting | 8 (18.6%) | | | | | | | 5 (11.6%) | | | | | |
|  | Diarrhea | 0 (0%) | | | | | | | 0 (0%) | | | | | |
|  | Liver dysfunction | 0 (0%) | | | | | | | 0 (0%) | | | | | |
|  | Renal dysfunction | 0 (0%) | | | | | | | 0 (0%) | | | | | |
|  | Peripheral neuropathy | 2 (4.7%) | | | | | | | 1 (2.3%) | | | | | |
|  | Hypertension | 0 (0%) | | | | | | | 0 (0%) | | | | | |
| Oza (2018) | Adverse Event (G3/4) | Intermittent linsitinib + paclitaxel | | | | Continuous linsitinib + paclitaxel | | | | | Paclitaxel | | | |
|  | Any treatment-related AE | 26 (52.0%) | | | | 16 (32.7%) | | | | | 14 (28.6%) | | | |
|  | Fatigue | 2 (4.0%) | | | | 1 (2.0%) | | | | | 1 (2.0%) | | | |
|  | Nausea | 0 | | | | 1 (2.0%) | | | | | 0 | | | |
|  | **Anemia** | 3 (6.0%) | | | | 0 | | | | | 3 (6.1%) | | | |
|  | **Diarrhea** | 1 (2.0%) | | | | 1 (2.0%) | | | | | 0 | | | |
|  | **Peripheral neuropathy** | 0 | | | | 1 (2.0%) | | | | | 2 (4.1%) | | | |
|  | Vomiting | 2 (4.0%) | | | | 2 (4.1%) | | | | | 0 | | | |
|  | Constipation | 0 | | | | 1 (2.0%) | | | | | 0 | | | |
|  | Nail disorder | 0 | | | | 1 (2.0%) | | | | | 1 (2.0%) | | | |
|  | **Neutropenia** | 10 (20.0%) | | | | 3 (6.1%) | | | | | 3 (6.1%) | | | |
|  | Asthenia | 1 (2.0%) | | | | 0 | | | | | 1 (2.0%) | | | |
|  | **Prolonged QT electrocardiogram** | 2 (4.0%) | | | | 0 | | | | | 0 | | | |
|  | Paresthesia | 1 (2.0%) | | | | 1 (2.0%) | | | | | 0 | | | |
|  | Mucosal inflammation | 0 | | | | 0 | | | | | 1 (2.0%) | | | |
|  | Abdominal pain | 1 (2.0%) | | | | 0 | | | | | 0 | | | |
|  | Hyperglycemia | 1 (2.0%) | | | | 3 (6.1%) | | | | | 0 | | | |
| Roque (2022) | Grade 3 | Ixabepilone monotherapy | | | | | | | Ixabepilone + Bevacizumab | | | | | |
|  | Blood and lymphatic system disorders | 1 | | | | | | | 0 | | | | | |
|  | Ear and labyrinth disorders | 0 | | | | | | | 1 | | | | | |
|  | Gastrointestinal disorders | 1 | | | | | | | 6 | | | | | |
|  | General disorders and administration site conditions | 1 | | | | | | | 0 | | | | | |
|  | Metabolism and nutrition disorders | 2 | | | | | | | 1 | | | | | |
|  | Nervous system disorders | 1 | | | | | | | 0 | | | | | |
|  | Renal and urinary disorders | 0 | | | | | | | 1 | | | | | |
|  | Respiratory, thoracic and mediastinal disorders | 0 | | | | | | | 0 | | | | | |
|  | Grade 4 | Ixabepilone monotherapy | | | | | | | Ixabepilone + Bevacizumab | | | | | |
|  | Infections and infestations | 1 | | | | | | | 0 | | | | | |
|  | Investigations | 1 | | | | | | | 0 | | | | | |
| McGuire (2018) | Grade ≥ 3 | Olaratumab + Liposomal Doxorubicin | | | | | | | Liposomal Doxorubicin | | | | | |
|  | Fatigue | 7 (11.3%) | | | | | | | 1 (1.6%) | | | | | |
|  | Rash | 3 (4.8%) | | | | | | | 5 (8.2%) | | | | | |
|  | Abdominal pain | 2 (3.2%) | | | | | | | 8 (13.1%) | | | | | |
|  | Neutropenia | 8 (12.9%) | | | | | | | 5 (8.2%) | | | | | |
|  | Nausea | 2 (3.2%) | | | | | | | 1 (1.6%) | | | | | |
|  | Constipation | 2 (3.2%) | | | | | | | 0 | | | | | |
|  | Vomiting | 3 (4.8%) | | | | | | | 6 (9.8%) | | | | | |
|  | Palmar-plantar erythrodysesthesia syndrome | 7 (11.3%) | | | | | | | 4 (6.6%) | | | | | |
|  | Diarrhea | 2 (3.2%) | | | | | | | 0 | | | | | |
|  | Back pain | 1 (1.6%) | | | | | | | 1 (1.6%) | | | | | |
|  | Abdominal distension | 2 (3.2%) | | | | | | | 2 (3.3%) | | | | | |
|  | Urinary tract infection | 0 | | | | | | | 2 (3.3%) | | | | | |
|  | Headache | 0 | | | | | | | 1 (1.6%) | | | | | |
|  | Anemia | 3 (4.8%) | | | | | | | 1 (1.6%) | | | | | |
|  | Dehydration | 3 (4.8%) | | | | | | | 2 (3.3%) | | | | | |
| Chekerov (2018) |  | Topotecan and sorafeni Grade 3 | | | Topotecan and sorafeni Grade 4 | | | | Topotecan and placebo Grade 3 | | | Topotecan and placebo Grade 4 | | |
|  | **All** | 33 (40%) | | | 45 (54%) | | | | 40 (45%) | | | 37 (42%) | | |
|  | **Haematological** | 25 (30%) | | | 43 (52%) | | | | 34 (38%) | | | 36 (40%) | | |
|  | Leucopenia | 38 (46%) | | | 19 (23%) | | | | 36 (40%) | | | 11 (12%) | | |
|  | Neutropenia | 10 (12%) | | | 36 (43%) | | | | 20 (22%) | | | 28 (31%) | | |
|  | Thrombocytopenia | 17 (20%) | | | 6 (7%) | | | | 13 (15%) | | | 7 (8%) | | |
|  | Anaemia | 11 (13%) | | | 1 (1%) | | | | 14 (16%) | | | 3 (3%) | | |
|  | Lymphopenia | 1 (1%) | | | 0 | | | | 1 (1%) | | | 0 | | |
|  | **Non-haematological** | 49 (59%) | | | 7 (8%) | | | | 39 (44%) | | | 11 (12%) | | |
|  | **Cardiovascular** | 1 (1%) | | | 0 | | | | 4 (4%) | | | 1 (1%) | | |
|  | Thrombosis or embolism | 1 (1%) | | | 0 | | | | 3 (3%) | | | 1 (1%) | | |
|  | **Coagulation** | 1 (1%) | | | 0 | | | | 1 (1%) | | | 0 | | |
|  | **Constitutional symptoms** | 13 (16%) | | | 2 (2%) | | | | 8 (9%) | | | 1 (1%) | | |
|  | Fatigue | 9 (11%) | | | 1 (1%) | | | | 4 (4%) | | | 0 | | |
|  | Weight loss | 0 | | | 0 | | | | 1 (1%) | | | 0 | | |
|  | **Dermatology** | 19 (23%) | | | 0 | | | | 0 | | | 0 | | |
|  | Alopecia | 0 | | | 0 | | | | 0 | | | 0 | | |
|  | Hand-foot skin reaction | 11 (13%) | | | 0 | | | | 0 | | | 0 | | |
|  | Other dermatological symptoms | 11 (13%) | | | 0 | | | | 0 | | | 0 | | |
|  | **Endocrine** | 1 (1%) | | | 0 | | | | 1 (1%) | | | 0 | | |
|  | **Gastrointestinal** | 19 (23%) | | | 1 (1%) | | | | 30 (34%) | | | 1 (1%) | | |
|  | Non-malignant ascites | 4 (5%) | | | 0 | | | | 6 (7%) | | | 0 | | |
|  | Constipation | 2 (2%) | | | 0 | | | | 5 (6%) | | | 0 | | |
|  | Ileus | 4 (5%) | | | 0 | | | | 8 (9%) | | | 0 | | |
|  | Nausea | 3 (4%) | | | 0 | | | | 9 (10%) | | | 0 | | |
|  | Vomiting | 2 (2%) | | | 0 | | | | 5 (6%) | | | 0 | | |
|  | Diarrhoea | 4 (5%) | | | 0 | | | | 4 (4%) | | | 0 | | |
|  | **Haemorrhage or bleeding** | 1 (1%) | | | 0 | | | | 1 (1%) | | | 0 | | |
|  | **Infection** | 12 (14%) | | | 2 (2%) | | | | 16 (18%) | | | 3 (3%) | | |
|  | Infection catheter related | 2 (2%) | | | 0 | | | | 6 (7%) | | | 0 | | |
|  | Febrile neutropenia | 4 (5%) | | | 2 (2%) | | | | 3 (3%) | | | 2 (2%) | | |
|  | Urinary tract infection | 0 | | | 0 | | | | 1 (1%) | | | 0 | | |
|  | Other infection | 6 (7%) | | | 0 | | | | 8 (9%) | | | 1 (1%) | | |
|  | **Lymphatics** | 0 | | | 0 | | | | 0 | | | 0 | | |
|  | Oedema | 0 | | | 0 | | | | 0 | | | 0 | | |
|  | **Metabolic or laboratory** | 14 (17%) | | | 1 (1%) | | | | 12 (13%) | | | 4 (4%) | | |
|  | Alanine aminotransferase | 2 (2%) | | | 0 | | | | 4 (4%) | | | 0 | | |
|  | Aspartate aminotransferase | 1 (1%) | | | 0 | | | | 3 (3%) | | | 0 | | |
|  | Creatinine | 0 | | | 0 | | | | 1 (1%) | | | 0 | | |
|  | γ-glutamyltransferase | 1 (1%) | | | 0 | | | | 2 (2%) | | | 1 (1%) | | |
|  | Hyperkalaemia | 2 (2%) | | | 1 (1%) | | | | 0 | | | 1 (1%) | | |
|  | Hypokalaemia | 4 (5%) | | | 0 | | | | 5 (6%) | | | 0 | | |
|  | Hyponatraemia | 4 (5%) | | | 0 | | | | 4 (4%) | | | 0 | | |
|  | **Neurology** | 4 (5%) | | | 0 | | | | 2 (2%) | | | 0 | | |
|  | Neuropathy | 3 (4%) | | | 0 | | | | 0 | | | 0 | | |
|  | **Pain** | 8 (10%) | | | 1 (1%) | | | | 8 (9%) | | | 1 (1%) | | |
|  | Abdominal pain | 5 (6%) | | | 0 | | | | 4 (4%) | | | 1 (1%) | | |
|  | **Pulmonary** | 10 (12%) | | | 1 (1%) | | | | 5 (6%) | | | 4 (4%) | | |
|  | Dyspnoea | 6 (7%) | | | 1 (1%) | | | | 3 (3%) | | | 2 (2%) | | |
|  | Pleural effusion | 2 (2%) | | | 0 | | | | 2 (2%) | | | 0 | | |
|  | **Renal or genitourinary** | 1 (1%) | | | 1 (1%) | | | | 3 (3%) | | | 0 | | |
| Marth (2017) | Adverse Event | PLD+Trebananib Grade 3 | | | PLD+Trebananib Grade 4 | | | | PLD+Placebo  Grade 3 | | | PLD+Placebo Grade 4 | | |
|  | Palmar-plantar erythrodysesthesia | 22 (20%) | | | 0 | | | | 13 (12%) | | | 0 | | |
|  | Localised oedema | 5 (4%) | | | 0 | | | | 2 (2%) | | | 0 | | |
|  | Nausea | 7 (6%) | | | 0 | | | | 5 (5%) | | | 0 | | |
|  | Fatigue | 8 (7%) | | | 0 | | | | 5 (5%) | | | 1 (1%) | | |
|  | Stomatitis | 7 (6%) | | | 1 (1%) | | | | 6 (6%) | | | 0 | | |
|  | Vomiting | 7 (6%) | | | 0 | | | | 6 (6%) | | | 0 | | |
|  | Abdominal pain | 7 (6%) | | | 0 | | | | 5 (5%) | | | 0 | | |
|  | Constipation | 2 (2%) | | | 0 | | | | 2 (2%) | | | 0 | | |
|  | Diarrhoea | 3 (3%) | | | 1 (1%) | | | | 5 (5%) | | | 0 | | |
|  | Ascites | 24 (21%) | | | 0 | | | | 7 (7%) | | | 0 | | |
|  | Rash | 2 (2%) | | | 0 | | | | 2 (2%) | | | 0 | | |
|  | Mucosal inflammation | 1 (1%) | | | 0 | | | | 2 (2%) | | | 0 | | |
|  | Decreased appetite | 1 (1%) | | | 0 | | | | 2 (2%) | | | 0 | | |
|  | Dyspnoea | 5 (4%) | | | 0 | | | | 3 (3%) | | | 2 (2%) | | |
|  | Hypokalaemia | 8 (7%) | | | 1 (1%) | | | | 2 (2%) | | | 1 (1%) | | |
|  | Neutropenia | 8 (7%) | | | 1 (1%) | | | | 13 (12%) | | | 4 (4%) | | |
|  | Dyspepsia | 1 (1%) | | | 0 | | | | 0 | | | 0 | | |
|  | Alopecia | 0 | | | 0 | | | | 0 | | | 0 | | |
|  | Pyrexia | 1 (1%) | | | 0 | | | | 0 | | | 0 | | |
|  | Back pain | 0 | | | 0 | | | | 0 | | | 0 | | |
|  | Abdominal pain, upper | 0 | | | 0 | | | | 2 (2%) | | | 0 | | |
|  | Headache | 3 (3%) | | | 0 | | | | 1 (1%) | | | 0 | | |
|  | Pleural effusion | 6 (5%) | | | 0 | | | | 4 (4%) | | | 1 (1%) | | |
|  | Dizziness | 0 | | | 0 | | | | 1 (1%) | | | 0 | | |
|  | Anaemia | 3 (3%) | | | 1 (1%) | | | | 4 (4%) | | | 0 | | |
|  | Oropharyngeal pain | 0 | | | 0 | | | | 0 | | | 0 | | |
|  | Asthenia | 0 | | | 0 | | | | 3 (3%) | | | 0 | | |
|  | Dry skin | 0 | | | 0 | | | | 0 | | | 0 | | |
|  | Weight decreased | 0 | | | 0 | | | | 2 (2%) | | | 0 | | |
|  | Nasopharyngitis | 0 | | | 0 | | | | 0 | | | 0 | | |
|  | Insomnia | 0 | | | 0 | | | | 1 (1%) | | | 0 | | |
|  | Hypertension | 3 (3%) | | | 0 | | | | 0 | | | 0 | | |
|  | Muscle spasms | 0 | | | 0 | | | | 0 | | | 0 | | |
|  | Peripheral neuropathy | 0 | | | 0 | | | | 0 | | | 0 | | |
|  | Abdominal distension | 0 | | | 0 | | | | 2 (2%) | | | 0 | | |
|  | Skin hyperpigmentation | 0 | | | 0 | | | | 0 | | | 0 | | |
|  | Pain in extremity | 0 | | | 0 | | | | 0 | | | 0 | | |
|  | Pruritus | 0 | | | 0 | | | | 0 | | | 0 | | |
|  | Upper respiratory tract infection | 1 (1%) | | | 0 | | | | 0 | | | 0 | | |
|  | Hypomagnesaemia | 4 (4%) | | | 0 | | | | 1 (1%) | | | 0 | | |
| Joly (2022) | Grade 3/4 | Paclitaxel + Pazopanib | | | | | | | Paclitaxel alone | | | | | |
|  | Hypertension | 35 (44%) | | | | | | | 3 (8%) | | | | | |
|  | Fatigue | 16 (20%) | | | | | | | 3 (8%) | | | | | |
|  | Anemia | 10 (13%) | | | | | | | 4 (11%) | | | | | |
|  | Diarrhea | 9 (11%) | | | | | | | 2 (5%) | | | | | |
|  | Hepatic cytolysis | 18 (23%) | | | | | | | 7 (19%) | | | | | |
|  | Lymphopenia | 9 (11%) | | | | | | | 4 (11%) | | | | | |
|  | Neutropenia | 22 (28%) | | | | | | | 8 (22%) | | | | | |
|  | Pain | 5 (6%) | | | | | | | 5 (14%) | | | | | |
|  | Abdominal pain | 5 (6%) | | | | | | | 3 (8%) | | | | | |
|  | Nausea | 5 (6%) | | | | | | | 1 (3%) | | | | | |
|  | Anorexia | 8 (10%) | | | | | | | 1 (3%) | | | | | |
|  | Vomiting | 8 (10%) | | | | | | | 4 (11%) | | | | | |
|  | Sensory neuropathy | 3 (4%) | | | | | | | 3 (8%) | | | | | |
|  | Thrombocytopenia | 7 (9%) | | | | | | | 4 (11%) | | | | | |
|  | Mucositis | 2 (3%) | | | | | | | 1 (3%) | | | | | |
|  | Constipation | 3 (4%) | | | | | | | 1 (3%) | | | | | |
|  | Infection | 5 (6%) | | | | | | | 2 (5%) | | | | | |
|  | Proteinuria | 3 (4%) | | | | | | | 0 | | | | | |
|  | Edema | 1 (1%) | | | | | | | 1 (3%) | | | | | |
|  | Dyspnea | 4 (5%) | | | | | | | 2 (5%) | | | | | |
|  | Creatinine increased | 2 (3%) | | | | | | | 0 | | | | | |
|  | Onycholysis | 0 | | | | | | | 2 (5%) | | | | | |
|  | Dyspepsia | 1 (1%) | | | | | | | 0 | | | | | |
|  | Bilirubin increased | 3 (4%) | | | | | | | 1 (3%) | | | | | |
|  | Skin rash | 0 | | | | | | | 0 | | | | | |
|  | Hand–foot syndrome | 0 | | | | | | | 0 | | | | | |
|  | Motor neuropathy | 0 | | | | | | | 0 | | | | | |
|  | Pulmonary embolism | 7 (9%) | | | | | | | 0 | | | | | |
|  | Intestinal obstruction | 5 (6%) | | | | | | | 3 (8%) | | | | | |
|  | Thrombosis | 1 (1%) | | | | | | | 0 | | | | | |
