# Supplementary material for: Molecular targeted therapy in combination with chemotherapy for the treatment of platinum-resistant/refractory ovarian cancer (PROC): a systematic review and network meta-analysis
Source: Ann Med. 2026 Feb 23;58(1):2624215. doi: 10.1080/07853890.2026.2624215 (PMC12931348; doi:10.1080/07853890.2026.2624215)
Supplement: Supplementary Table S2.docx [file IANN_A_2624215_SM0122.docx]

Supplementary Table 2A League table of the Bayesian network meta-analysis for PFS

| CTRL |  |  |  |  |  |  |  |  |  |  |  |  |  |  |  |
| --- | --- | --- | --- | --- | --- | --- | --- | --- | --- | --- | --- | --- | --- | --- | --- |
| 1.82 (1.13, 2.91) | ADA |  |  |  |  |  |  |  |  |  |  |  |  |  |  |
| 1.28 (0.88, 1.86) | 0.71 (0.39, 1.29) | AVE |  |  |  |  |  |  |  |  |  |  |  |  |  |
| 1.75 (1.01, 3.01) | 0.96 (0.47, 1.98) | 1.37 (0.71, 2.64) | BER |  |  |  |  |  |  |  |  |  |  |  |  |
| 1.94 (1.63, 2.3) | 1.07 (0.65, 1.77) | 1.51 (1, 2.28) | 1.11 (0.63, 1.96) | BEV |  |  |  |  |  |  |  |  |  |  |  |
| 0.83 (0.52, 1.33) | **0.46 (0.24, 0.89)** | 0.65 (0.36, 1.18) | **0.48 (0.23, 0.98)** | **0.43 (0.26, 0.71)** | LIN |  |  |  |  |  |  |  |  |  |  |
| 0.96 (0.64, 1.44) | **0.53 (0.29, 0.98)** | 0.75 (0.44, 1.3) | 0.55 (0.28, 1.08) | **0.5 (0.32, 0.77)** | 1.16 (0.62, 2.13) | OLA |  |  |  |  |  |  |  |  |  |
| 1.37 (1.14, 1.65) | 0.75 (0.45, 1.25) | 1.07 (0.71, 1.62) | 0.78 (0.44, 1.39) | **0.71 (0.55, 0.91)** | 1.65 (0.99, 2.72) | 1.42 (0.92, 2.21) | PAZ |  |  |  |  |  |  |  |  |
| 1.43 (1.08, 1.9) | 0.79 (0.46, 1.37) | 1.12 (0.7, 1.79) | 0.82 (0.44, 1.51) | 0.74 (0.53, 1.03) | 1.72 (0.99, 2.96) | 1.49 (0.91, 2.43) | 1.04 (0.75, 1.47) | PER |  |  |  |  |  |  |  |
| 1 (0.65, 1.54) | 0.55 (0.29, 1.04) | 0.78 (0.44, 1.38) | 0.57 (0.29, 1.15) | **0.52 (0.32, 0.82)** | 1.2 (0.63, 2.27) | 1.04 (0.58, 1.87) | 0.73 (0.46, 1.17) | 0.7 (0.42, 1.17) | SAR |  |  |  |  |  |  |
| 0.97 (0.7, 1.35) | **0.53 (0.3, 0.95)** | 0.76 (0.46, 1.25) | 0.55 (0.29, 1.05) | **0.5 (0.35, 0.73)** | 1.17 (0.66, 2.06) | 1.01 (0.6, 1.69) | 0.71 (0.48, 1.04) | 0.68 (0.44, 1.05) | 0.97 (0.56, 1.67) | SER |  |  |  |  |  |
| 1.67 (1.2, 2.32) | 0.92 (0.52, 1.63) | 1.3 (0.79, 2.13) | 0.95 (0.51, 1.8) | 0.86 (0.59, 1.25) | 2 (1.13, 3.54) | 1.73 (1.03, 2.91) | 1.22 (0.83, 1.78) | 1.16 (0.75, 1.8) | 1.67 (0.97, 2.87) | 1.72 (1.07, 2.73) | SOR |  |  |  |  |
| 0.97 (0.72, 1.32) | **0.53 (0.31, 0.94)** | 0.76 (0.47, 1.23) | 0.55 (0.3, 1.04) | **0.5 (0.35, 0.71)** | 1.16 (0.67, 2.04) | 1.01 (0.61, 1.68) | 0.71 (0.49, 1.01) | 0.68 (0.45, 1.03) | 0.97 (0.57, 1.64) | 1 (0.64, 1.57) | **0.58 (0.37, 0.91)** | TREB |  |  |  |
| 1.92 (1.29, 2.85) | 1.06 (0.57, 1.96) | 1.5 (0.87, 2.59) | 1.1 (0.56, 2.16) | 0.99 (0.65, 1.53) | 2.31 (1.25, 4.26) | 1.99 (1.14, 3.5) | 1.4 (0.9, 2.17) | 1.34 (0.82, 2.18) | 1.92 (1.07, 3.45) | 1.98 (1.19, 3.31) | 1.15 (0.69, 1.92) | 1.98 (1.2, 3.26) | TREM |  |  |
| 1.59 (1.04, 2.43) | 0.87 (0.46, 1.66) | 1.24 (0.71, 2.18) | 0.91 (0.45, 1.81) | 0.82 (0.52, 1.29) | 1.91 (1.01, 3.59) | 1.65 (0.93, 2.95) | 1.16 (0.73, 1.84) | 1.11 (0.67, 1.85) | 1.59 (0.87, 2.92) | 1.64 (0.95, 2.8) | 0.95 (0.56, 1.63) | 1.63 (0.97, 2.77) | 0.83 (0.46, 1.48) | VIN |  |
| 1.19 (0.94, 1.5) | 0.66 (0.39, 1.11) | 0.93 (0.6, 1.44) | 0.68 (0.38, 1.23) | **0.61 (0.46, 0.82)** | 1.43 (0.85, 2.4) | 1.23 (0.78, 1.96) | 0.87 (0.64, 1.17) | 0.83 (0.58, 1.2) | 1.19 (0.73, 1.95) | 1.23 (0.82, 1.83) | 0.71 (0.48, 1.07) | 1.23 (0.83, 1.8) | **0.62 (0.39, 0.98)** | 0.75 (0.46, 1.22) | VIS |

Supplementary Table 2B League table of the Bayesian network meta-analysis for OS

| CTRL |  |  |  |  |  |  |  |  |  |  |  |  |  |  |
| --- | --- | --- | --- | --- | --- | --- | --- | --- | --- | --- | --- | --- | --- | --- |
| 1.78 (1.11, 2.88) | ADA |  |  |  |  |  |  |  |  |  |  |  |  |  |
| 1.12 (0.87, 1.45) | 0.63 (0.36, 1.08) | AVE |  |  |  |  |  |  |  |  |  |  |  |  |
| 1.38 (1.16, 1.63) | 0.77 (0.46, 1.29) | 1.23 (0.9, 1.67) | BEV |  |  |  |  |  |  |  |  |  |  |  |
| 0.91 (0.61, 1.36) | **0.51 (0.27, 0.95)** | 0.81 (0.5, 1.3) | 0.66 (0.43, 1.02) | LIN |  |  |  |  |  |  |  |  |  |  |
| 0.91 (0.59, 1.41) | **0.51 (0.27, 0.97)** | 0.81 (0.49, 1.34) | 0.66 (0.41, 1.05) | 1 (0.55, 1.81) | OLA |  |  |  |  |  |  |  |  |  |
| 1.21 (0.93, 1.56) | 0.68 (0.39, 1.16) | 1.07 (0.75, 1.55) | 0.88 (0.64, 1.19) | 1.33 (0.83, 2.13) | 1.33 (0.8, 2.2) | PAZ |  |  |  |  |  |  |  |  |
| 1.33 (0.95, 1.84) | 0.74 (0.41, 1.33) | 1.18 (0.78, 1.79) | 0.96 (0.67, 1.39) | 1.46 (0.87, 2.44) | 1.46 (0.84, 2.52) | 1.1 (0.72, 1.67) | PER |  |  |  |  |  |  |  |
| 1.06 (0.63, 1.78) | 0.59 (0.29, 1.21) | 0.94 (0.53, 1.69) | 0.77 (0.45, 1.33) | 1.17 (0.61, 2.24) | 1.17 (0.59, 2.3) | 0.88 (0.49, 1.57) | 0.8 (0.43, 1.48) | SAR |  |  |  |  |  |  |
| 1.01 (0.63, 1.61) | 0.57 (0.29, 1.1) | 0.9 (0.53, 1.53) | 0.73 (0.45, 1.2) | 1.11 (0.6, 2.05) | 1.11 (0.59, 2.1) | 0.84 (0.49, 1.42) | 0.76 (0.43, 1.35) | 0.95 (0.47, 1.9) | SER |  |  |  |  |  |
| 1.54 (1.07, 2.21) | 0.86 (0.47, 1.57) | 1.37 (0.88, 2.13) | 1.12 (0.75, 1.67) | 1.69 (0.99, 2.89) | 1.69 (0.96, 2.99) | 1.28 (0.82, 1.99) | 1.16 (0.71, 1.9) | 1.45 (0.77, 2.73) | 1.52 (0.84, 2.76) | SOR |  |  |  |  |
| 1.06 (0.72, 1.57) | 0.6 (0.32, 1.1) | 0.95 (0.6, 1.51) | 0.77 (0.51, 1.18) | 1.17 (0.67, 2.04) | 1.17 (0.65, 2.1) | 0.88 (0.55, 1.4) | 0.8 (0.48, 1.34) | 1.01 (0.53, 1.91) | 1.05 (0.58, 1.93) | 0.69 (0.41, 1.18) | TREB |  |  |  |
| 1.56 (0.88, 2.77) | 0.88 (0.41, 1.85) | 1.39 (0.74, 2.61) | 1.14 (0.62, 2.06) | 1.72 (0.85, 3.45) | 1.72 (0.83, 3.54) | 1.3 (0.69, 2.43) | 1.18 (0.61, 2.29) | 1.48 (0.68, 3.19) | 1.55 (0.73, 3.26) | 1.02 (0.51, 2.01) | 1.47 (0.73, 2.93) | TREM |  |  |
| 1.16 (0.75, 1.8) | 0.65 (0.34, 1.25) | 1.03 (0.62, 1.72) | 0.84 (0.53, 1.35) | 1.28 (0.7, 2.3) | 1.28 (0.69, 2.38) | 0.96 (0.58, 1.6) | 0.88 (0.51, 1.51) | 1.09 (0.55, 2.16) | 1.15 (0.61, 2.18) | 0.75 (0.42, 1.34) | 1.09 (0.61, 1.96) | 0.74 (0.36, 1.54) | VIN |  |
| 0.83 (0.62, 1.1) | **0.46 (0.27, 0.81)** | 0.74 (0.5, 1.08) | **0.6 (0.43, 0.83)** | 0.91 (0.56, 1.48) | 0.91 (0.54, 1.53) | 0.68 (0.47, 1) | **0.62 (0.4, 0.97)** | 0.78 (0.43, 1.41) | 0.82 (0.47, 1.42) | **0.54 (0.34, 0.85)** | 0.78 (0.48, 1.25) | 0.53 (0.28, 1.01) | 0.71 (0.42, 1.2) | VIS |

Supplementary Table 2C League table of the Bayesian network meta-analysis for AEs

| CTRL |  |  |  |  |  |  |  |  |  |  |  |  |  |  |  |
| --- | --- | --- | --- | --- | --- | --- | --- | --- | --- | --- | --- | --- | --- | --- | --- |
| **0.56 (0.36, 0.77)** | ADA |  |  |  |  |  |  |  |  |  |  |  |  |  |  |
| **0.74 (0.56, 0.97)** | 1.32 (0.85, 2.19) | AVE |  |  |  |  |  |  |  |  |  |  |  |  |  |
| 1.06 (0.48, 2.36) | 1.91 (0.8, 4.72) | 1.44 (0.62, 3.35) | BER |  |  |  |  |  |  |  |  |  |  |  |  |
| **0.78 (0.67, 0.9)** | 1.4 (0.97, 2.2) | 1.06 (0.78, 1.45) | 0.74 (0.33, 1.65) | BEV |  |  |  |  |  |  |  |  |  |  |  |
| 0.67 (0.38, 1.07) | 1.2 (0.62, 2.25) | 0.9 (0.49, 1.57) | 0.63 (0.24, 1.59) | 0.86 (0.48, 1.41) | LIN |  |  |  |  |  |  |  |  |  |  |
| 1.1 (0.83, 1.46) | 1.98 (1.27, 3.27) | 1.49 (1.01, 2.22) | 1.04 (0.44, 2.41) | 1.41 (1.03, 1.94) | 1.65 (0.95, 3.1) | OLA |  |  |  |  |  |  |  |  |  |
| **0.59 (0.47, 0.72)** | 1.06 (0.7, 1.69) | 0.8 (0.56, 1.13) | 0.56 (0.24, 1.26) | **0.75 (0.58, 0.98)** | 0.88 (0.52, 1.62) | **0.53 (0.37, 0.76)** | PAZ |  |  |  |  |  |  |  |  |
| 0.96 (0.8, 1.15) | 1.72 (1.18, 2.73) | 1.3 (0.94, 1.81) | 0.91 (0.4, 2.04) | 1.23 (0.97, 1.56) | 1.44 (0.86, 2.6) | 0.87 (0.62, 1.22) | 1.63 (1.23, 2.18) | PER |  |  |  |  |  |  |  |
| 0.85 (0.45, 1.47) | 1.52 (0.74, 3.07) | 1.15 (0.58, 2.15) | 0.79 (0.29, 2.11) | 1.08 (0.56, 1.92) | 1.27 (0.57, 2.81) | 0.77 (0.39, 1.44) | 1.44 (0.74, 2.62) | 0.88 (0.46, 1.59) | SAR |  |  |  |  |  |  |
| 0.85 (0.65, 1.08) | 1.52 (0.99, 2.49) | 1.15 (0.79, 1.67) | 0.8 (0.35, 1.84) | 1.09 (0.8, 1.45) | 1.27 (0.74, 2.35) | 0.77 (0.52, 1.12) | 1.44 (1.03, 2) | 0.88 (0.64, 1.2) | 1 (0.54, 1.99) | SER |  |  |  |  |  |
| 0.92 (0.83, 1.02) | 1.65 (1.17, 2.55) | 1.25 (0.94, 1.69) | 0.87 (0.39, 1.93) | 1.18 (0.99, 1.41) | 1.38 (0.85, 2.46) | 0.84 (0.62, 1.13) | 1.57 (1.24, 2.01) | 0.96 (0.78, 1.18) | 1.09 (0.62, 2.08) | 1.08 (0.83, 1.45) | SOR |  |  |  |  |
| 0.94 (0.8, 1.1) | 1.68 (1.16, 2.65) | 1.27 (0.93, 1.76) | 0.89 (0.39, 1.99) | 1.2 (0.97, 1.49) | 1.4 (0.85, 2.53) | 0.85 (0.62, 1.18) | 1.6 (1.23, 2.1) | 0.98 (0.77, 1.25) | 1.11 (0.62, 2.13) | 1.1 (0.83, 1.51) | 1.02 (0.84, 1.23) | TREB |  |  |  |
| **0.22 (0.01, 0.88)** | 0.39 (0.02, 1.72) | 0.29 (0.02, 1.24) | 0.2 (0.01, 1.06) | 0.28 (0.02, 1.14) | 0.32 (0.02, 1.5) | **0.2 (0.01, 0.83)** | 0.37 (0.02, 1.55) | **0.22 (0.01, 0.93)** | 0.25 (0.01, 1.2) | 0.25 (0.01, 1.07) | **0.23 (0.01, 0.97)** | **0.23 (0.01, 0.95)** | TREM |  |  |
| **0.71 (0.51, 0.91)** | 1.26 (0.8, 2.09) | 0.96 (0.64, 1.4) | 0.66 (0.28, 1.54) | 0.9 (0.64, 1.22) | 1.05 (0.6, 1.98) | **0.64 (0.42, 0.94)** | 1.2 (0.82, 1.69) | 0.74 (0.51, 1.01) | 0.83 (0.44, 1.66) | 0.83 (0.56, 1.21) | 0.77 (0.55, 1.01) | 0.75 (0.53, 1.02) | 3.27 (0.76, 56.44) | VIN |  |
| 0.89 (0.58, 1.37) | 1.61 (0.93, 2.92) | 1.21 (0.73, 2.02) | 0.84 (0.34, 2.07) | 1.14 (0.73, 1.8) | 1.34 (0.7, 2.71) | 0.81 (0.49, 1.35) | 1.52 (0.94, 2.47) | 0.93 (0.59, 1.48) | 1.06 (0.52, 2.26) | 1.05 (0.64, 1.74) | 0.97 (0.62, 1.51) | 0.95 (0.6, 1.5) | 4.19 (0.92, 73.35) | 1.27 (0.77, 2.13) | VIS |
