# Supplementary material for: Molecular targeted therapy in combination with chemotherapy for the treatment of platinum-resistant/refractory ovarian cancer (PROC): a systematic review and network meta-analysis
Source: Ann Med. 2026 Feb 23;58(1):2624215. doi: 10.1080/07853890.2026.2624215 (PMC12931348; doi:10.1080/07853890.2026.2624215)
Supplement: Supplementary Table S6.docx [file IANN_A_2624215_SM0121.docx]

**Supplementary Table S6.** Reason of “high-risk” grading in risk of bias assessment.

| Study | Reason of “high-risk” grading |
| --- | --- |
| Duska 2019 | **Blinding of participants and personnel:**  The trial was conducted as an open-label study without blinding of participants or study personnel, which may introduce performance bias |
| Joly 2022 | **Blinding of participants and personnel:**  The trial was conducted as an open-label study without blinding of participants or study personnel, which may introduce performance bias |
| Konstantinopoulos 2020 | **Blinding of participants and personnel:**  The study was conducted as an open-label trial without blinding of participants or study personnel, which may introduce performance bias |
| Lee 2022 | **Allocation concealment:**  Although randomization was centrally managed, the trial report did not clearly describe specific concealment measures (e.g., sealed envelopes or automated systems), leaving a risk of selection bias.  **Blinding of participants and personnel:**  The study was conducted as an open-label trial without blinding of participants or study personnel, which may introduce performance bias |
| Liu 2016 | **Blinding of participants and personnel:**  The trial was conducted as an open-label study without blinding of participants or study personnel, which may introduce performance bias |
| McGuire 2018 | **Blinding of participants and personnel:**  The study was conducted as an open-label trial without blinding of participants or study personnel, which may introduce performance bias |
| Oza 2018 | **Allocation concealment:**  The study only mentioned central randomization but did not provide details on concealment measures; without clear safeguards such as IVRS/IWRS or sealed opaque envelopes, investigators may have been able to foresee assignments, introducing a high risk of selection bias.  **Blinding of participants and personnel:**  The trial was conducted as an open-label study without blinding of participants or investigators, which may introduce performance bias |
| Pignata 2016 | **Blinding of participants and personnel:**  The study was conducted as an open-label trial without blinding of participants or study personnel, which may introduce performance bias |
| Pujade-Lauraine 2014 | **Blinding of participants and personnel:**  The study was conducted as an open-label trial without blinding of participants or study personnel, which may introduce performance bias |
| Pujade-Lauraine 2021 | **Blinding of participants and personnel:**  The study was conducted as an open-label trial without blinding of participants or study personnel, which may introduce performance bias |
| Sharma 2021 | **Blinding of participants and personnel:**  The study was conducted as an open-label trial without blinding of participants or study personnel, which may introduce performance bias |
| Shoji 2021 | **Blinding of participants and personnel:**  The study was conducted as an open-label trial without blinding of participants or study personnel, which may introduce performance bias |
