# Supplementary material for: Molecular targeted therapy in combination with chemotherapy for the treatment of platinum-resistant/refractory ovarian cancer (PROC): a systematic review and network meta-analysis
Source: Ann Med. 2026 Feb 23;58(1):2624215. doi: 10.1080/07853890.2026.2624215 (PMC12931348; doi:10.1080/07853890.2026.2624215)
Supplement: Supplementary Table S3.docx [file IANN_A_2624215_SM0120.docx]

**Supplementary Table S3.** Reasons of excluded studies in full-text screening.

| Study |  | Reasons of exclusion |
| --- | --- | --- |
| Vanderstichele 2022 | DOI: 10.1016/j.ygyno.2022.01.034 | **Inconsistent inclusion and exclusion criteria:**  Excluded because the trial compared olaparib monotherapy versus chemotherapy, whereas our NMA only included RCTs assessing targeted therapy in combination with chemotherapy versus chemotherapy. In addition, the study population was not limited to PROC patients. |
| Trillsch 2016 | DOI: 10.1093/annonc/mdw236 | **Inconsistent inclusion and exclusion criteria:**  Exploratory subgroup analysis of the AURELIA trial; not an independent RCT comparing targeted therapy + chemotherapy versus chemotherapy. |
| Su 2009 |  | **Inconsistent inclusion and exclusion criteria:**  Pilot study, not a phase II/III RCT; small sample size. |
| Pujade-Lauraine 2016 | DOI: 10.1200/JCO.2015.62.1474 | **Inconsistent inclusion and exclusion criteria:**  olasertib monotherapy vs chemotherapy; not a targeted therapy + chemotherapy trial. |
| Penson 2020 | DOI: 10.1200/JCO.19.02745 | **Inconsistent inclusion and exclusion criteria:**  Platinum-sensitive population and olaparib monotherapy vs chemotherapy; not a targeted therapy + chemotherapy trial. |
| Nasu 2022 | DOI: 10.1007/s10147-021-02103-7 | **Inconsistent inclusion and exclusion criteria:**  Single-arm/observational design with small sample size; not a phase II/III RCT. |
| Musacchio 2021 | DOI: 10.1136/ijgc-2021-002593 | **Inconsistent inclusion and exclusion criteria:**  Investigational regimen was niraparib + dostarlimab (PARPi + ICI), not targeted therapy + chemotherapy. |
| Pujade-Lauraine 2018 | DOI: 10.2217/fon-2018-0070 | **Inconsistent inclusion and exclusion criteria:**  Description of the randomized phase III trial design evaluating the role of checkpoint inhibition in women with ovarian cancer. |
| Herzog 2023 | DOI: 10.2217/fon-2023-0246 | **Inconsistent inclusion and exclusion criteria:**  ngoing phase III trial; immunotherapy (Nemvaleukin ± Pembrolizumab) vs chemotherapy;no results available |
| Hamanishi 2021 | DOI: 10.1200/JCO.21.00334 | **Inconsistent inclusion and exclusion criteria:**  Nivolumab monotherapy vs chemotherapy; not a targeted therapy + chemotherapy trial. |
| Colombo 2022 | DOI: 10.1016/j.ygyno.2022.01.015 | **Inconsistent inclusion and exclusion criteria:**  Cediranib + Olaparib (dual targeted therapy) vs chemotherapy; not targeted therapy + chemotherapy. |
| Colombo 2012 | DOI: 10.1200/jco.2011.38.8082 | **Inconsistent inclusion and exclusion criteria:**  Patupilone (chemotherapy) vs PLD (chemotherapy); not a targeted therapy + chemotherapy trial. |
| Banerjee 2018 | DOI: 10.1093/annonc/mdy023 | **Inconsistent inclusion and exclusion criteria:**  Lifastuzumab vedotin (ADC) monotherapy vs PLD; not a targeted therapy + chemotherapy trial. |
| Arend 2021 | DOI: 10.1016/j.ygyno.2021.02.014 | **Inconsistent inclusion and exclusion criteria:**  The intervention was targeted anti-cancer gene therapy: ofranergene obadenovec  (VB-111) |
| Michael Eichbaum 2011 | DOI: 10.1186/1471-2407-11-453 | **Inconsistent inclusion and exclusion criteria:**  This article is a study protocol without reported clinical outcomes |
| Zhang 2016 |  | **Inconsistent inclusion and exclusion criteria:**  Single-center retrospective study; not a prospective RCT. |
| Fotopoulou 2014 | DOI: 10.1093/annonc/mdt515 | **Inconsistent inclusion and exclusion criteria:**  This study evaluated phenoxodiol (a biomodulator/chemosensitizer) combined with carboplatin rather than a standard targeted agent, and therefore did not meet our inclusion criteria. |
| Lee 2018 | DOI: 10.1016/j.ygyno.2018.10.031 | **Inconsistent inclusion and exclusion criteria:**  This was a real-world retrospective observational study rather than a randomized controlled trial and was therefore excluded. |
| González-Martín 20116 | DOI: 10.1097/IGC.0000000000000695 | **Inconsistent inclusion and exclusion criteria:**  Prospective, single-arm study (non-randomized) of pertuzumab plus chemotherapy in PROC |
| Nicoletta Colombo | DOI: 10.1016/S1359-6349(15)70004-2 | **Inconsistent inclusion and exclusion criteria:**  The intervention was trabectedin, a cytotoxic chemotherapy agent, which does not meet the inclusion criteria. |
| Khalfaoui KE 2012 |  | **Full text unavailable** |
| MIRV Is Safe and Efficacious in FRalpha-High, Platinum-Resistant Ovarian Cancer | DOI: 10.1158/2159-8290.CD-RW2023-022 | **Full text unavailable** |
| McGuire 2010 |  | **Full text unavailable** |
| Kurzeder 2014 |  | **Full text unavailable** |
| Rebecca C Arend 2021 | DOI: 10.1016/j.ygyno.2021.02.014 | **Incinsistent study objectives:**  This study is a preplanned interim futility analysis of the OVAL trial, reporting only CA-125 response rates without final clinical outcomes such as PFS or OS. |
| Su 2009 |  | **Incinsistent study objectives:**  reporting only CA-125 response rates without final clinical outcomes such as PFS or OS. |
| Martin R Stockler | DOI: 10.1200/JCO.2013.51.4240 | **Incinsistent study objectives:**  not an independent RCT; no additional OS, PFS, or grade 3–4 AE data. |
| Stockler 2014 | DOI: 10.1200/JCO.2013.51.4240 | **Incinsistent study objectives:**  Secondary PRO analysis of the AURELIA trial; not an independent RCT; no additional OS, PFS, or grade 3–4 AE data. |
| Madariaga 2022 | DOI: 10.1016/j.ygyno.2022.08.006 | **Incinsistent study objectives:**  Exploratory PRO-CTCAE substudy; reported only patient-reported symptomatic AEs (e.g., swallowing difficulty, diarrhea, fatigue) without OS/PFS or grade 3–4 AE outcomes |
| Banerjee 2023 |  | **Duplications:**  Excluded due to data overlap with the included Banerjee 2022 publication. |
|  |  |  |
| Liu 2016 | DOI: 10.1200/JCO.2016.67.1891 | **Duplications:**  This study is duplicated with the included articles |
| Lheureux 2021 | DOI: 10.1016/S0140-6736(20)32554-X | **Duplications:**  This study is duplicated with the included articles |
| McNeish 2014 | DOI: 10.1093/annonc/mdu363 | **Duplications:**  This study is duplicated with the included articles |
